# Rapid evolution of an adaptive taste polymorphism disrupts courtship behavior

**DOI:** 10.1038/s42003-022-03415-8

**Published:** 2022-05-12

**Authors:** Ayako Wada-Katsumata, Eduardo Hatano, Samantha McPherson, Jules Silverman, Coby Schal

**Affiliations:** Department of Entomology and Plant Pathology and W.M. Keck Center for Behavioral Biology, North Carolina State University, Raleigh, North Carolina 27695 USA

**Keywords:** Behavioural ecology, Sexual selection

## Abstract

The evolution of adaptive behavior often requires changes in sensory systems. However, rapid adaptive changes in sensory traits can adversely affect other fitness-related behaviors. In the German cockroach, a gustatory polymorphism, ‘glucose-aversion (GA)’, supports greater survivorship under selection with glucose-containing insecticide baits and promotes the evolution of behavioral resistance. Yet, sugars are prominent components of the male’s nuptial gift and play an essential role in courtship. Behavioral and chemical analyses revealed that the saliva of GA females rapidly degrades nuptial gift sugars into glucose, and the inversion of a tasty nuptial gift to an aversive stimulus often causes GA females to reject courting males. Thus, the rapid emergence of an adaptive change in the gustatory system supports foraging, but it interferes with courtship. The trade-off between natural and sexual selection under human-imposed selection can lead to directional selection on courtship behavior that favors the GA genotype.

## Abstract (web summary)

A study of German cockroaches with glucose-aversion reveals how this adaptation to avoid glucose-containing insecticides causes females to reject courting males, because their salivary enzymes degrade sugars in the male’s nuptial gift into glucose.

## Introduction

Adaptive traits are shaped by environmental pressures that select on natural genetic variation. Generally, traits evolve slowly over a long time^1,2^, and as natural selection compels evolutionary novelty in the sensory system, sexual selection may constrain these changes, and vice versa^3,4^. On the other hand, under intense human-imposed selection such as agricultural, aquacultural and pest control interventions, adaptive traits can evolve rapidly and sweep through populations, even if they incur some reproductive costs^5^. For example, *flatwing* mutants of the Pacific field cricket (*Teleogryllus oceanicus*) emerged in males under strong selection by the invasive parasitic fly *Ormia ochracea*, which locates singing male crickets and lays its eggs on them^6,7^. The *flatwing* mutation is adaptive because males evade parasitoids, but the loss of acoustic sexual signaling also reduces their reproductive success. An alternative mating strategy has emerged in *flatwing* males – they position themselves near singing males and intercept females attracted to the calling males^8^. The *Teleogryllus*–*Ormia* system represents a trade-off between sexual selection (favoring reproductive success) and natural selection (favoring survival)^9^. Such trade-offs have been documented in visual and acoustic sexual communication, and rapid directional evolution is often imposed by invasive predators or parasitoids. However, such trade-offs between natural and sexual selection, rapid directional changes in sexual communication, and evidence of these processes in ‘real-time’ have not been well documented in reproductive systems that rely heavily on chemoreception.

We employed the German cockroach *Blattella germanica* as a compelling system in which foraging success and mating success are differentially affected by a recently evolved gustatory polymorphism. Gustatory receptor neurons (GRNs) in the mouthparts of the cockroach detect sugars, such as glucose, maltose and maltotriose, and mediate both foraging and sexual communication^10,11^. During courtship^12^, males expose specialized tergal glands^13–15^ and offer females an oligosaccharide-rich secretion that includes maltose and maltotriose, as well as phospholipids, cholesterol and various amino acids^16–23^. By attracting the female to his highly palatable secretion, the male places the female in the proper position for copulation and lowers her behavioral threshold to mate, thus gaining competitive advantage in female mate choice^10,12^. The female must mount the male and feed on the nuptial secretion long enough for the male to extend his abdomen under the female and engage her genitalia; short nuptial feeding results in interrupted and often failed courtship. Mating success is thus maximized through the convergence of the quality of the male’s nuptial secretion and the female’s gustatory sensitivity to it^12^.

As a perennial household pest with significant public health, social and economic impacts^24^, the German cockroach has been the target of intense human-imposed selection with sugar-containing insecticide baits. In response, multiple cockroach populations rapidly evolved behavioral resistance to these baits in the form of glucose-aversion (GA) — GRNs in the mouthparts detect glucose as a deterrent rather than phagostimulant and drive an avoidance behavior that enables cockroaches to elude the toxic bait^11,25,26^. The GA trait follows Mendelian-like inheritance patterns^27^. Because it is highly adaptive in the presence of glucose-containing toxic baits, the GA genotype can rapidly replace glucose-accepting wild-type (WT) cockroaches^28^. This unique sensory polymorphism is an excellent model of rapid evolution of chemosensory-based behavior in the anthropogenic environment^29^. Recently, we observed that GA females have lower mating success with WT males than do WT females, but the underlying behavioral mechanisms were unclear^30^. We also found that salivary glucosidases of both WT and GA females rapidly digest oligosaccharides in food, resulting in lower sugar consumption in GA females but not in WT females^31^. In this study, we hypothesized that salivary digestion of the nuptial secretion might release glucose, disrupting nuptial feeding of GA females but not WT females, and possibly explaining lower mating success in GA females. To identify behavioral events that mediate successful and failed courtship sequences, we compared seven behavioral parameters in courtship of WT and GA females. By artificially manipulating the nuptial secretion in the male tergal glands, we tested whether the quality of the nuptial secretion intercedes in successful and failed courtship sequences. We also generated recombinant lines of WT and GA cockroaches to confirm an association between the GA trait and rejection of the nuptial secretion. Finally, we tested whether saliva could rapidly digest oligosaccharides in the nuptial secretion, releasing glucose and thus transforming a tasty nuptial gift to an aversive stimulus. Overall, we demonstrate and discuss how glucose-aversion imposes a trade-off by favoring survivorship under natural selection in the presence of toxic baits, but it disrupts courtship under sexual selection. Rapid directional changes in the sexual communication system are expected to correct for the lower reproductive success of GA females.

## Results

### Interrupted nuptial feeding reduces mating success of GA females

**Fig. 1:**
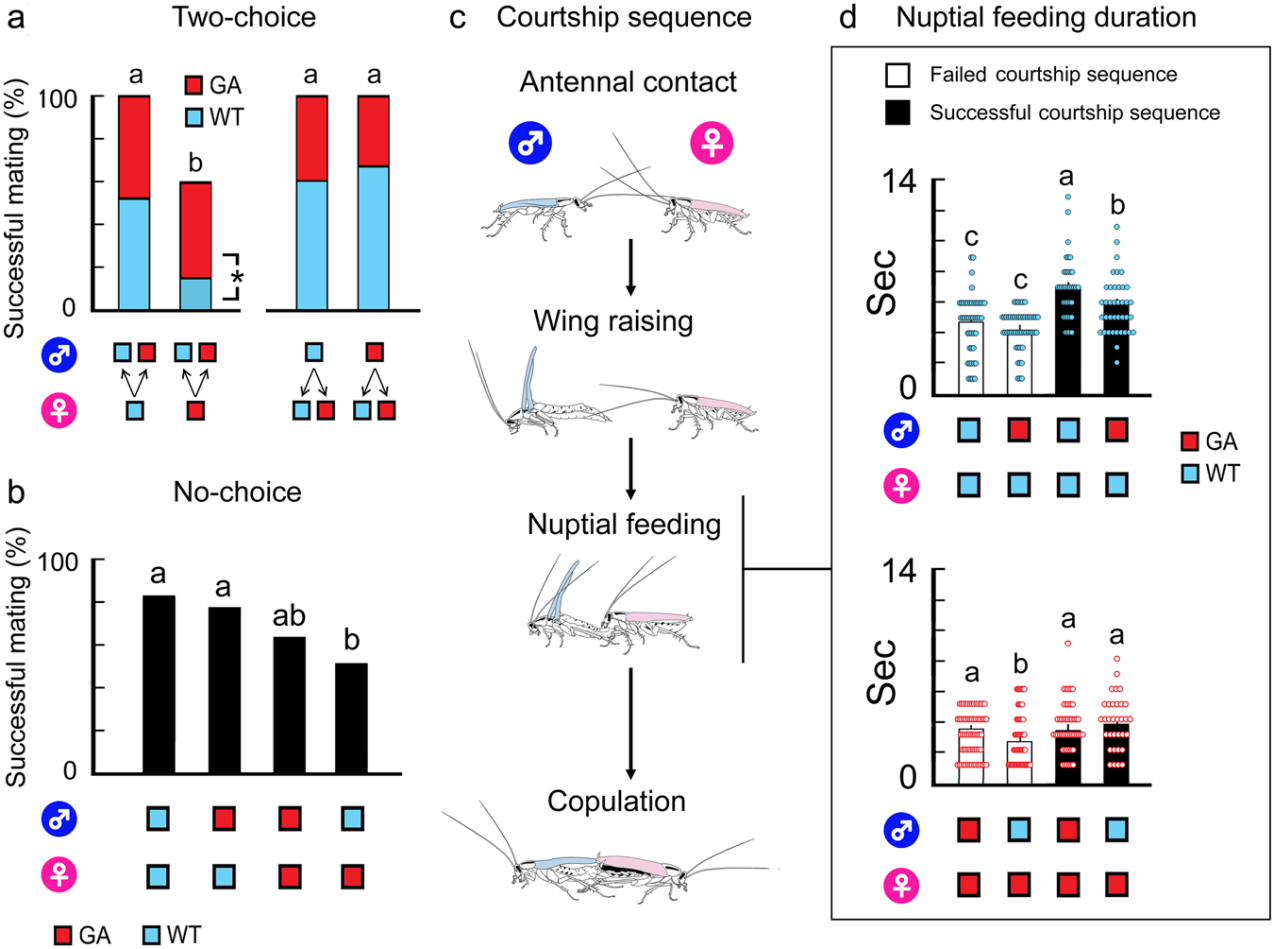
Lower mating success of GA females is associated with short nuptial feeding. **a** Two-choice mating assay showing lower mating success in GA females. GA females experienced more courtship failures with WT males than with GA males. Different letters indicate significant differences in mating success (χ^2^_(3)_ = 29.50, *P* < 0.001). The asterisk indicates a significant difference in mating success of WT and GA males paired with GA females (χ^2^_(1)_ = 5.40, *P* = 0.020). **b** No-choice mating assays showing lower mating success in GA females than WT females. Different letters indicate significant differences in mating success (χ^2^_(3)_ = 16.70, *P* = 0.0008). **c** Courtship sequence of *B. germanica*, highlighting the importance of female nuptial feeding on the male’s tergal secretion (original drawings by A.W-K.). **d** Nuptial feeding duration (mean ± SE) is the time that a female spent exploring the male’s tergal gland with her mouthparts. The durations of failed and successful courtship sequences are indicated by white and black bars, respectively. Different letters indicate significant differences among treatments (ANOVA, Tukey’s HSD test, *F*_(3, 167)_ = 18.97, *P* < 0.0001 (upper); *F*_(3, 222)_ = 3.69, *P* = 0.01 (lower)). Short nuptial feeding duration causes failure to copulate in both WT and GA females and GA females had significantly shorter nuptial feeding than did WT females (Supplementary Table 2).

In choice tests with a GA female and two males, WT males had significantly lower mating success than GA males, whereas both WT and GA males experienced similar mating success with WT females (Fig. 1a left, Supplementary Table 1). In choice assays with a male and two females (Fig. 1a right), there was no significant difference in mating success between GA and WT females when assayed with either a WT or GA male. In no-choice tests (Fig. 1b, Supplementary Table 2), GA females experienced significantly lower mating success than WT females when paired with WT males, but there was no significant difference in mating success in the other three pairings (WT Male/WT Female, GA Male/WT Female, GA Male/GA Female). Because this deficit in GA females was a consequence of behaviors expressed during courtship, we analyzed key behavioral events that correlate with mating success in both strains (Fig. 1c). Comparative behavioral analysis of successful and failed courtship sequences revealed significant differences between WT and GA females in the parameter associated with ‘nuptial feeding’ (Fig. 1d, Supplementary Table 2). In failed courtship sequences, WT females engaged in a single nuptial feeding event of short duration (4.2–4.6 s) (Fig. 1d upper). On the other hand, in successful courtship sequences WT females mounted males 1.4–1.7 times (nuptial feeding events), and the duration of a single successful nuptial feeding event was significantly longer than in failed courtship sequences (5.8–6.9 s; ANOVA, *F*_(3, 167)_ = 18.97, *P* < 0.0001).

These findings suggest an association of failure to copulate with short nuptial feeding and that successful copulation requires that females engage in protracted nuptial feeding to enable the male to grasp the female’s genitalia. Additionally, nuptial feeding duration was significantly longer in pairings of WT Male/WT Female than GA Male/WT Female, suggesting that other courtship traits affected the duration of nuptial feeding in WT females. In contrast, GA females had significantly shorter nuptial feeding than WT females in both failed and successful courtship sequences (2.8–3.6 and 3.5–3.8 s, respectively), and no significant difference was found in the durations of failed and successful sequences. Although courtship sequences of GA females with WT males had intermittent nuptial feeding, they significantly increased the number of nuptial feeding events (2.7 events, Supplementary Table 2) and extended the total nuptial feeding duration in successful sequences (9.2 s, Supplementary Table 2). In contrast, in failed sequences of GA females the total nuptial feeding duration remained short (2.8 s, Supplementary Table 2). These results reveal that behaviors unique to GA females interrupt nuptial feeding, cause short feeding duration, and result in failure to mate. Additionally, it is important to note that GA females mated successfully with GA males (Fig. 1a, b), without engaging in more nuptial feeding events and despite their relatively short total nuptial feeding duration (4.8 s, Fig. 1d). It appears that the pair of GA cockroaches have a different courtship strategy which does not rely on the females’ GA trait to improve mating success. In this study we focused on the mechanisms that underlie short nuptial feeding of GA females that lead to failure to mate with WT males. Ongoing studies are investigating the mechanisms that enable successful copulation in GA cockroach pairs.

### Short nuptial feeding duration is mediated by the quality of the nuptial secretion

**Fig. 2:**
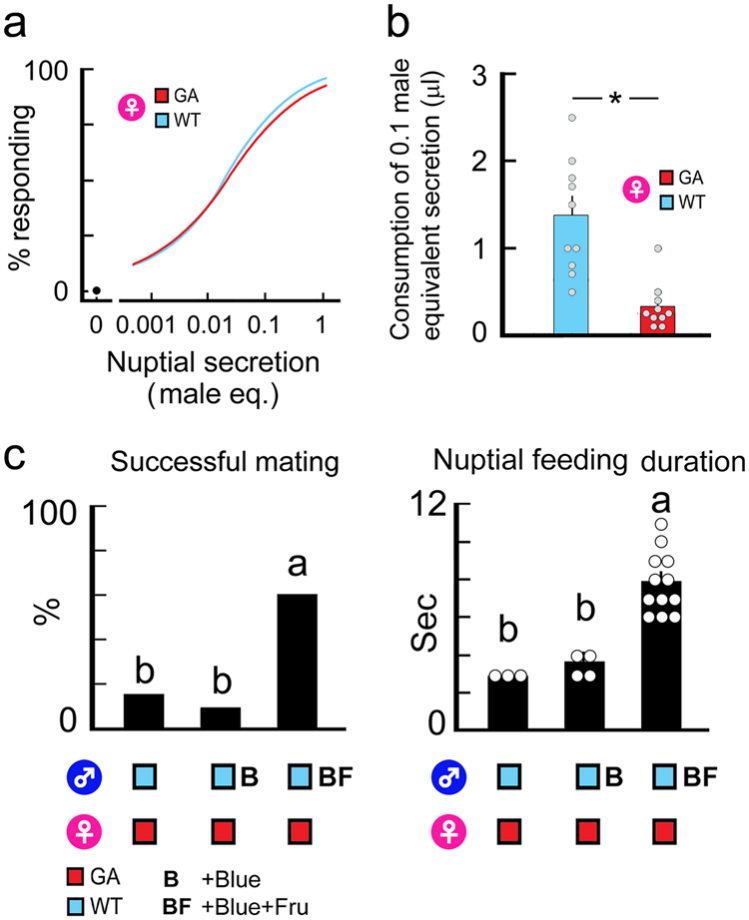
Mismatch between the quality of male nuptial secretion and female gustatory preferences causes short nuptial feeding bouts and lower mating success in GA females. **a** Dose-feeding response curves for WT and GA females in response to WT male nuptial secretion represented in male-equivalents (*n* = 20 for each). None of the females responded to water (0 male-equivalents, black circle), the vehicle used for nuptial secretion. The EC_50_ values (95% CI) (male equivalents (eq.)) were 0.021 (0.015, 0.027) for WT females and 0.026 (0.018, 0.033) for GA females. **b** Consumption of the WT male nuptial secretion by WT and GA females in a single feeding bout (mean ± SE, *n* = 10 each). The asterisk indicates a significant difference (*t* = 4.56, *P* = 0.0002). **c** Pairing of GA females with intact WT males (Control, *n* = 20) and with WT males whose nuptial secretion was augmented with blue dye (B, *n* = 25) or blue dye and fructose (BF, *n* = 20) (mean ± SE). The addition of fructose to the tergal gland reservoirs of WT males significantly increased their mating success (χ^2^_(2)_ = 13.23, *P* < 0.001). GA females engaged in longer nuptial feeding bouts (nuptial feeding duration) (ANOVA, Tukey’s HSD test, *F*_(2, 16)_ = 23.96, *P* < 0.0001). Different letters indicate significant differences among treatments.

To understand what causes the short nuptial feeding of GA females, we performed bioassays that measured the female’s initial instantaneous response to tastants (Acceptance-Rejection assay)^31^. Both WT and GA females were equally stimulated to initiate nuptial feeding by the tergal gland secretions of WT males (Fig. 2a, Supplementary Table 3). However, in Consumption assays^31^, which measured the total amount eaten in a single feeding bout, GA females ingested significantly less nuptial secretion than WT females (Fig. 2b, Supplementary Table 3). These results suggested the presence of a mismatch between the composition of the nuptial secretion and the taste preferences of GA females, and that feeding by GA females was suspended after they initially accepted the nuptial secretion, resulting in failure to mate.

In addition, because GA females also consume less maltose and maltotriose than WT females^31^, we tested whether longer nuptial feeding and successful mating of GA females could be restored by increasing the quality of the tergal secretion of WT males with fructose, which is not aversive to GA cockroaches (Fig. 2c, Supplementary Table 4)^11,26,31^. Mating success was low in unmanipulated pairs, with short nuptial feeding by GA females (Chi-square test, χ^2^_(2)_ = 14.94, *P* < 0.001). However, the addition of fructose to the tergal secretion of WT males increased the nuptial feeding duration of GA females, resulting in significantly higher mating success. These results confirm that longer nuptial feeding leads to successful mating, and it can be restored in GA females by modifying the composition of the male’s tergal secretion. They further indicate that components of the nuptial secretion disrupt nuptial feeding of GA females and that the GA trait features prominently in this maladaptive outcome.

### Genetic association of the GA trait with short nuptial feeding

**Fig. 3:**
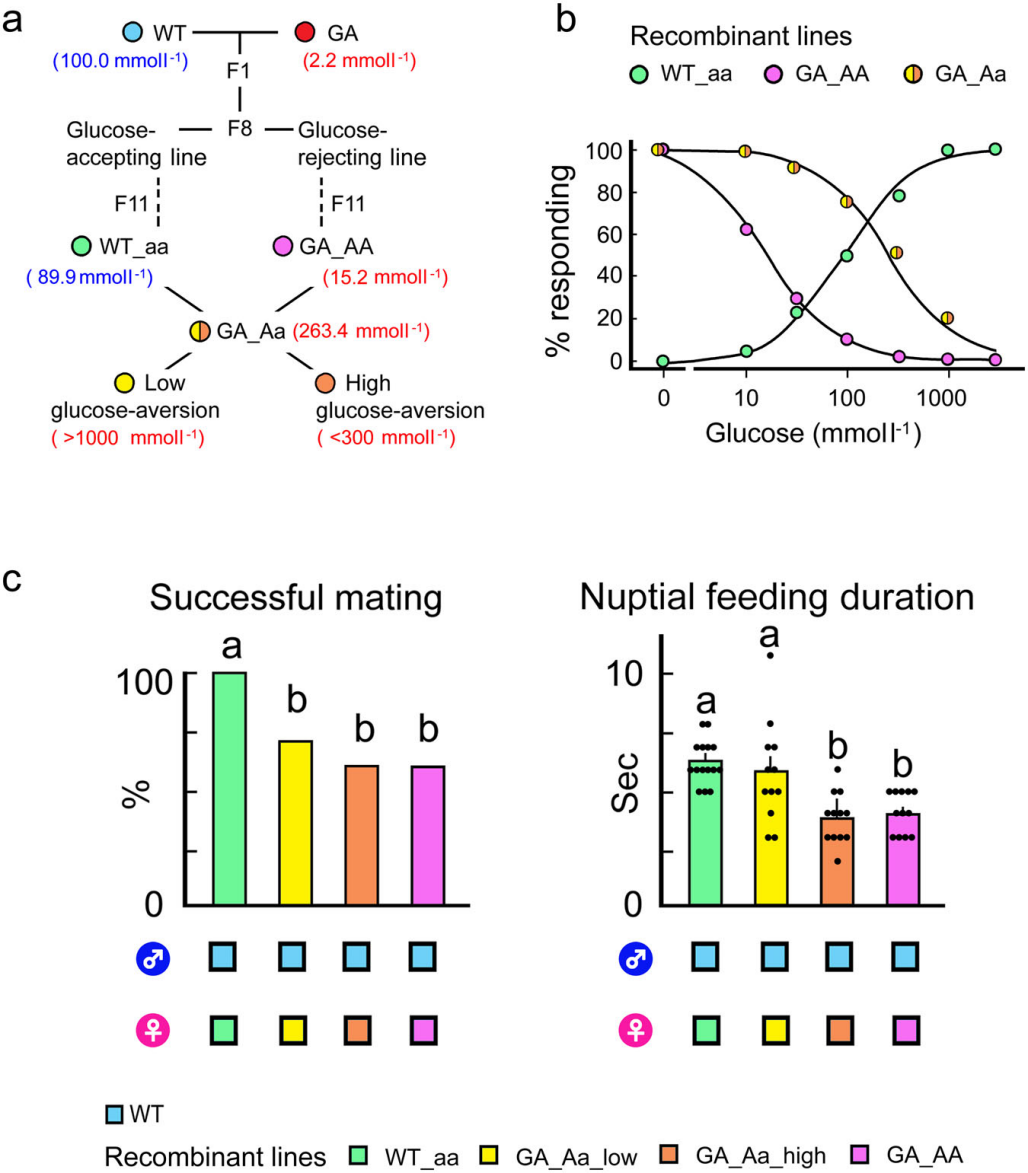
Recombinant cockroach lines confirm the association of interrupted nuptial feeding in females with the glucose-aversion trait. **a** The experimental design used to generate four recombinant lines. The EC_50_ values for glucose acceptance in WT and WT_aa cockroaches (Blue), and glucose rejection in GA, GA_Aa and GA_AA cockroaches (Red) are shown in parentheses (*n* > 16 each). **b** Dose-feeding response curves for glucose in females of three recombinant lines. Because of Mendelian-like segregation of the GA trait, all GA_Aa cockroaches (heterozygous for the GA trait) were glucose-averse, but their sensitivity to glucose was lower than in GA_AA cockroaches. WT_aa cockroaches accepted glucose in a dose-dependent manner. **c** In no-choi**c**e mating assays using WT males paired with females from the four types of recombinant lines, GA females with high sensitivity to glucose (GA_Aa_high and GA_aa) had significantly lower mating success (left, *n* > 15 each, χ^2^(3) = 8.45, *P* = 0.018) and shorter nuptial feeding duration (right, ANOVA, Tukey’s HSD test, *F*_(2, 14)_ = 17.65, *P* = 0.0001), indicating that short nuptial feeding associates with the GA trait. Different letters indicate significant differences among treatments.

To determine if the GA trait intercedes in the short nuptial feeding, we generated a recombinant line of WT and GA cockroaches to homogenize their genetic backgrounds and thus created three genotypes: WT_aa, GA_AA and GA_Aa (Fig. 3a). Dose-response curves using the Acceptance-Rejection assay showed that WT_aa females accepted glucose in a dose-dependent manner, whereas GA_AA and GA_Aa females rejected glucose; the effective glucose concentration that elicited aversion in 50% (EC_50_) of GA_AA females was lower than in GA_Aa females (Fig. 3b, Supplementary Table 5). This result is consistent with our previous findings^27^ that feeding responses of GA females may be mediated by not only Mendelian inheritance but also by unknown factors that are either genetically linked with and/or unrelated to the GA trait. To test the association of sensitivity to glucose with courtship parameters, we separated the GA_Aa females into two groups: GA_Aa_low females displayed low sensitivity to glucose and rejected only high concentrations of glucose (>1000 mmol l^−1^), while GA_Aa_high females were highly sensitive to glucose and rejected it at lower concentrations (100–300 mmol l^−1^) (Fig. 3a, b). In no-choice mating assays with WT males GA_AA and GA_Aa_high females had significantly lower mating success than GA_Aa_low and WT_aa females (Fig. 3c). Although each nuptial feeding event was of short duration, these females extended their total nuptial feeding duration with more nuptial feeding events, while GA_Aa_low females behaved similarly to WT_aa females (Supplementary Table 6). These results indicate that the heritable GA trait is associated with interrupted nuptial feeding in GA females.

### Mechanisms responsible for short nuptial feeding in GA females

**Fig. 4:**
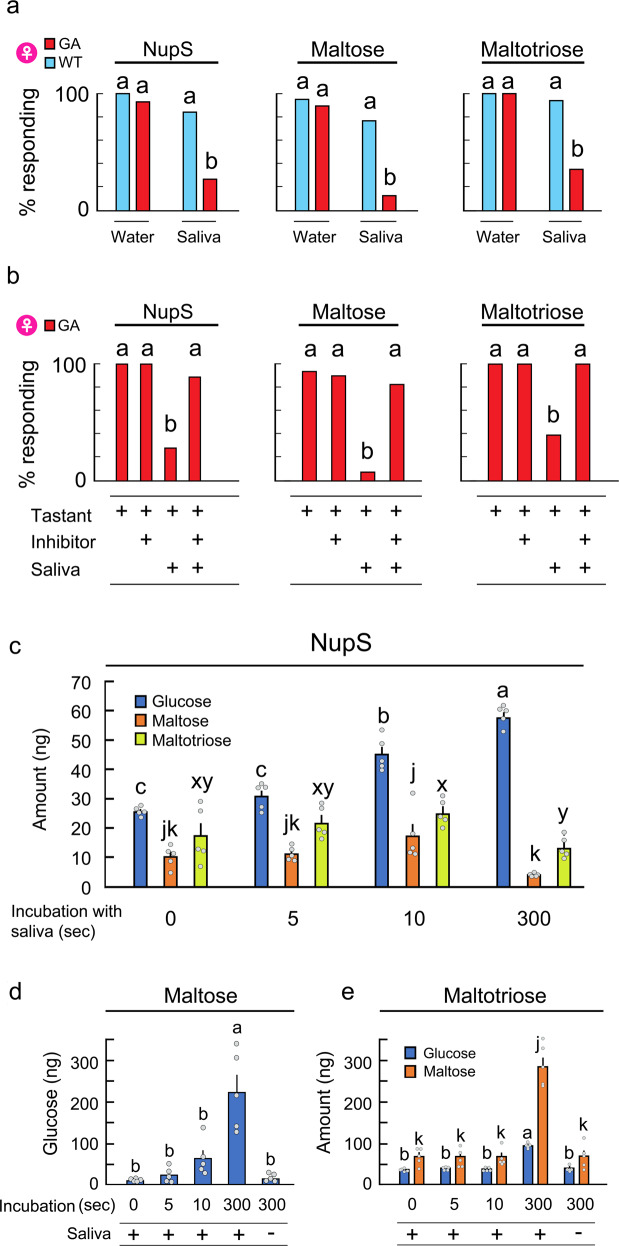
Saliva hydrolyzes nuptial secretion and oligosaccharides, releasing glucose, which disrupts courtship by interrupting nuptial feeding of glucose-averse females. **a** Saliva of GA females, mixed with WT nuptial secretion (NupS), maltose or maltotriose, interrupts acceptance of the nuptial secretion by GA females but not WT females. Different letters indicate significant differences (*n* > 20 each, Chi-square test with Holm’s method, *P* < 0.05, See Supplementary Table 7 for *P* values). **b** Addition of glucosidase inhibitor (acarbose) to saliva restored acceptance by GA females of previously rejected WT nuptial secretion, maltose and maltotriose, demonstrating the contribution of salivary digestion to the rejection of glucose-containing sugars. Different letters indicate significant differences (*n* > 20 each, Chi-square test with Holm’s method, *P* < 0.05, See Supplementary Table 8 for *P* values). **c**–**e** Time-course of sugar accumulation when 1 male-equivalent of WT male nuptial secretion, maltose or maltotriose was incubated with 1 µl of GA female saliva. Addition of saliva increased the glucose concentration in all mixtures, indicating that glucose transformed the taste quality of the nuptial secretion to a distasteful deterrent for GA females. Different letters indicate significant differences (*n* = 5 each, Mean ± SE, ANOVA, Tukey’s HSD test, *P* < 0.05, See Supplementary Table 9 and Table 10 for *P-*values).

Previous studies using dissected tergites that included whole tergal glands documented the presence of oligosaccharides composed of glucose, including maltose and maltotriose, but there was no indication that free glucose was a component of the secretion^18–23^. This is consistent with other studies, showing that GA females and males accept various oligosaccharides, including maltose and maltotriose^26,31^. Thus, it was unclear how GA females instantaneously perceive and reject the nuptial secretion. In a previous study, we documented that salivary alpha-glucosidases in both WT and GA females hydrolyze dietary oligosaccharides to glucose, decreasing the acceptance of oligosaccharides in GA females, but not in WT females^31^. Therefore, we suspected that the same mechanism might result in rejection of the nuptial secretion by GA females. We collected the secretion directly from the tergal gland reservoirs of WT males and added either WT or GA female saliva to it, as well as to maltose or maltotriose. All three mixes were accepted by GA females, but at a significantly lower rate than by WT females (Fig. 4a Supplementary Table 7). Co-incubation of saliva with acarbose, an inhibitor of glycoside hydrolase enzymes in cockroach saliva^31^, confirmed that salivary alpha-glucosidases were highly effective at reducing acceptance by GA females of maltose, maltotriose and male nuptial secretion (Fig. 4b, Supplementary Table 8).

GC-MS analysis revealed that the tergal secretion contained maltose and maltotriose, and small amounts of glucose (Fig. 4c, Supplementary Table 9). Addition of saliva to either maltose or maltotriose produced glucose, and more glucose was released with longer incubation times (Fig. 4d, e, Supplementary Table 10). In incubations of maltose with 1 µl of GA female saliva, glucose increased 2-fold within 5 s, and another 10-fold after 300 s (Fig. 4d). In contrast, only 3-fold more glucose and 5-fold more maltose were released from maltotriose after a 300 s incubation, indicating that saliva hydrolyzes maltose more efficiently than maltotriose (Fig. 4e). When WT tergal secretion was incubated with GA female saliva, glucose increased significantly between 5 and 10 s of incubation (Fig. 4c), which is a critical period of nuptial feeding that correlates with successful copulation (Fig. 1d). The amounts of maltose and maltotriose decreased during the 300 s incubation with saliva. Notably, the nuptial secretion contains various oligosaccharides composed of glucose^18–23^, so glucose was likely released from other sugars as well. These results indicate that tergal secretion is rapidly hydrolyzed by female saliva during nuptial feeding, resulting in the deterioration of its taste quality for GA females. Thus, the short nuptial feeding and interrupted courtship of GA females are mediated by distasteful glucose that was released via salivary hydrolysis of the nuptial secretion.

## Discussion

We demonstrated that a change in taste valence is adaptive in foraging but interferes with courtship. New phenotypes that rapidly evolve under strong selection can create mismatches and conflicts in their adaptive values in different behavioral contexts^32^. For example, in *Drosophila melanogaster*, the DDT resistance allele is associated with phenotypes that have lower male mating success, smaller body size and lower aggressive performance^33^. As already discussed in the Introduction, the *flatwing* mutation generates trade-offs between survival and mating success in male crickets^6–8^.

**Fig. 5:**
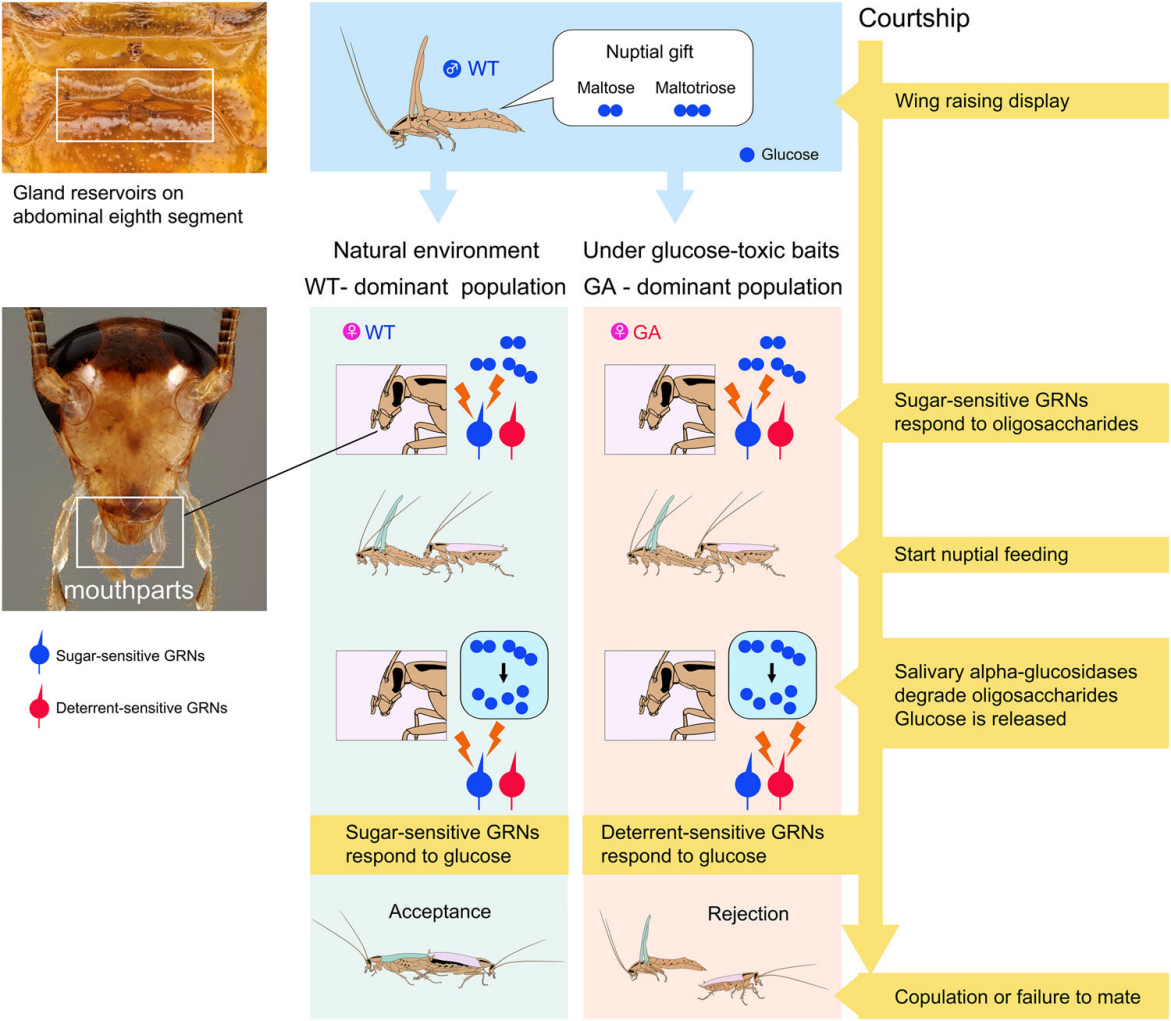
Summary illustrating the divergent effects of a gustatory polymorphism on survival and sexual behavior. Maltose and maltotriose contained in the male’s nuptial secretion (upper left) stimulate the female’s mouthparts (lower left) and lure the female to mount the courting male. Initially, both WT and GA females accept the secretion and commence nuptial feeding because oligosaccharides stimulate sugar-sensitive GRNs. However, during nuptial feeding saliva is secreted, salivary alpha-glucosidases hydrolyze the oligosaccharides, and free glucose is released. Glucose stimulates deterrent-sensitive GRNs of GA females, resulting in interrupted nuptial feeding. In contrast, glucose is highly appetitive to WT females, and they continue to feed on the nuptial secretion; longer nuptial feeding enables the male to grasp the female’s genitalia and copulate. Thus, the GA trait is highly adaptive for cockroach survival in the presence of glucose-containing toxic baits, but it interferes with sexual signaling by the male and results in lower mating success of GA females, especially with WT males.

Using the German cockroach as a model system, we tested whether similar tradeoffs may be evident with gustatory traits – did the rapid evolution of glucose-aversion adversely affect the gustation-dependent courtship system? Fine-scale analysis of the courtship sequences of WT, GA and recombinant lines of cockroaches demonstrated that nuptial feeding in GA females is interrupted into short feeding bouts, resulting in frequent failures of courting males to grasp the female genitalia and mate. Chemical analysis of male tergal secretions and quantitative bioassays of feeding by females revealed that the taste quality of nuptial secretion components conflicts with the emergent gustatory preferences of GA females. The nuptial gifting strategy likely evolved in the context of male exploitation of the female’s gustatory food preferences and her motivation to feed as her oocytes mature^10,34,35^. Therefore, the rapid transformation of glucose from appetitive to aversive stimulus resulted in an acute mismatch between the quality of the male’s nuptial gift and taste preferences of GA females. The mechanism that underlies this mismatch is the rapid processing of the male’s nuptial secretion by the female’s saliva. We found that the concentration of glucose in nuptial secretion (4.7 mmol l^−1^ in WT males, Supplementary Table 9) is not sufficient to stimulate its rejection by GA females, especially considering that glucose is embedded in a mix of phagostimulatory amino acids and lipids^16–23^. Indeed, GA females initially accepted the nuptial secretion, as did WT females (Fig. 2a). However, as previously shown^31^, saliva of WT and GA cockroaches contain alpha-glucosidases that hydrolyze oligosaccharides into alpha-glucose. In this study, as we predicted, salivary glucosidases rapidly released glucose during nuptial feeding, interrupting the feeding of GA females but not WT females. The released glucose activates deterrent-sensitive GRNs and suppresses the activation of sugar-sensitive GRNs in GA females^11^. Fig. 5 summarizes how this mechanism mediates short feeding durations and low consumption in GA females but not in WT females.

Our observations suggest that both GA males and GA females might express emergent courtship-related traits that mitigate their lower mating success. Considering GA male adaptations, we found that despite their short nuptial feeding duration, GA males had greater success in mating with GA females than did WT males (Fig. 1b, d). Moreover, GA males that mated with WT females did so despite the preceding nuptial feeding event being significantly shorter than when WT females were courted by WT males (Fig. 1d). These results suggest that elements of the courtship behavior of GA males differ from those WT males. Similar to the emergence of alternative male mate-finding strategies in *flatwing* crickets^6–9^, under persistent selection pressure by the gustatory preferences of GA females, GA males might express compensatory adaptive courtship traits such as more frequent approaches of females, short latency to copulation attempts, and changes in their nuptial gift components. Considering emergent adaptations of GA females, their saliva had 2-fold less alpha-glucosidase activity than the saliva of WT females^31^. It is possible that lower alpha-glucosidase activity of GA female saliva and the GA trait were jointly selected by sugar-containing toxic baits and the male’s nuptial gift. Our ongoing research is examining the full array of courtship traits of GA cockroaches and whether these traits are genetically linked to the GA trait.

It is imperative that we understand the evolution and spread of insecticide and behavioral resistance to inform ecologically sound pest management strategies. The origin of the GA trait is unknown, and it is also unclear how it introgresses and is maintained in various field populations^25^. We propose that assortative mating may have far-reaching implications for driving the GA trait into cockroach populations. Our results suggest that in mixed populations of GA and WT cockroaches, GA males may rapidly introgress the GA alleles into the WT population more than GA females because GA males efficiently mate with both genotypes, whereas GA females assortatively mate with GA males. Considering the population-wide changes that followed the emergence of the *flatwing* mutation in crickets^6–9^, selection by sugar-containing toxic baits would cause the eradication of the WT and heterozygous GA cockroaches, and their rapid replacement by homozygous GA cockroaches.

In summary, we previously elucidated how an adaptive gustatory trait can rapidly emerge under human-imposed ‘natural’ selection^25,28^ and in this study, we demonstrated that the emergent trait creates mismatches in sexual communication. Overall, pre-oral and oral processing of oligosaccharides by cockroach saliva dramatically extends the phenotypic range of the glucose-aversion genotype in both foraging and sexual contexts. Glucose-aversion thus has created trade-offs among fitness-related behaviors and compelled the rapid evolution of alternative compensatory reproductive tactics.

## Methods

### Cockroach strains

All cockroaches were maintained on rodent diet (Purina 5001, PMI Nutrition International, St. Louis, MO) and distilled water at 27 °C, ~40% RH, and a 12:12 h L:D cycle. The WT colony (Orlando Normal) was collected in Florida in 1947 and has served as a standard insecticide-susceptible strain. The GA colony (T-164) was collected in 1989, also in Florida, and shown to be aversive to glucose; continued artificial selection with glucose-containing toxic bait fixed the homozygous GA trait in this population (approximately 150 generations as of 2020).

### Generating recombinant lines and life history data

To homogenize the genetic backgrounds of the WT and GA strains, two recombinant colonies were initiated in 2013 by crossing 10 pairs of WT♂ × GA♀ and 10 pairs of GA♂ × WT♀ (Fig. 3a). At the F8 generation (free bulk mating without selection), 400 cockroaches were tested in two-choice feeding assays (see below) that assessed their initial response to tastants, as described in previous studies^11,26^. The cockroaches were separated into glucose-accepting and glucose-rejecting groups by the rapid Acceptance-Rejection assay (described in Feeding Bioassays). These colonies were bred for three more generations, and 200 cockroaches from each group were assayed in the F11 generation and backcrossed to obtain homozygous glucose-accepting (aa) and glucose-averse (AA) lines. Similar results were obtained in both directions of the cross, confirming previous findings of no sex linkage of the GA trait^27^. These two lines were defined as WT_aa (homozygotes, glucose-accepting) and GA_AA (homozygotes, glucose-averse). To obtain heterozygous GA cockroaches, GA_Aa, a single intercross group was generated from crosses of 10 pairs of WT_aa♂ × GA_AA♀ and 10 pairs of GA_AA♂ × WT_aa♀.

The GA trait follows Mendelian inheritance. Therefore, we used backcrosses, guided by two-choice feeding assays and feeding responses in Acceptance-rejection assays, to determine the homozygosity of WT and GA cockroaches. The cross of WT♂ × WT♀ produced homozygous F1 cockroaches showing maximal glucose-acceptance. The cross of GA♂ × GA♀ produced homozygous F1 cockroaches showing maximal glucose-aversion. The cross of WT × GA produced F1 heterozygotes with intermediate glucose-aversion. When the F1 heterozygotes were backcrossed with WT cockroaches, they produced F2 cockroaches with a 1:1 ratio of WT and GA phenotypes.

The two-choice feeding assay assessed whether cockroaches accepted or rejected glucose (binary: yes-no). Insects were held for 24 h without water, or starved without food and water. Either 10 adults or 2 day-old first instar siblings (30–40) were placed in a Petri dish (either 90 mm or 60 mm diameter × 15 mm height). Each Petri dish contained two agar discs: one disc contained 1% agar and 1 mmol l^−1^ red food dye (Allura Red AC), and the second disc contained 1% agar, 0.5 mmol l^−1^ blue food dye (Erioglaucine disodium salt) and either 1000 mmol l^−1^ or 3000 mmol l^−1^ glucose. The assay duration was 2 h during the dark phase of the insects’ L:D cycle. After each assay, the color of the abdomen of each cockroach was visually inspected under a microscope to infer the genotype.

We assessed whether the recombinant colonies had different traits from the parental WT and GA lines. We paired single newly eclosed females (day 0) with single 10–12 days-old males of the same line in a Petri dish (90 mm diameter, 15 mm height) with fresh distilled water in a 1.5 ml microcentrifuge tube and a pellet of rodent food, and monitored when they mated. When females formed egg cases, each gravid female was placed individually in a container (95 × 95 × 80 mm) with food and water until the eggs hatched. After removing the female, her offspring were monitored until adult emergence. We recorded the time to egg hatch, first appearance of each nymphal stage, first appearance of adults and the end of adult emergence. The first instar nymphs and adults in each cohort were counted to obtain measures of survivorship. Although there were significant differences in some of these parameters across all four strains, we found no significant differences between the two recombinant lines, except mating success, which was significantly lower in GA_AA♀ than WT_aa♀ (Supplementary Table 11).

### Mating bioassays

All mating sequences were recorded using an infra-red-sensitive camera (Polestar II EQ610, Everfocus Electronics, New Taipei City, Taiwan) coupled to a data acquisition board and analyzed by searchable and frame-by-frame capable software (NV3000, AverMedia Information) at 27 °C, ~40% RH and a 12:12 h L:D cycle. For behavioral analysis, tested pairs were classified into two groups: mated (successful courtship) and not-mated (failed courtship). Four distinct behavioral events (Fig. 1c, Contact, Wing raising, Nuptial feeding, and Copulation) were analyzed using seven behavioral parameters as shown in Supplementary Table 2.

We extracted behavioral data from successful courtship sequences, defined as courtship that led to Copulation. For failed courtship sequences, we extracted the behavioral data from the first courtship of both mated and not-mated groups, because most pairs in both groups failed to copulate in their first encounter, and there were no significant differences in behavioral parameters between the two groups.

To assay female choice, we conducted two-choice mating assays (Fig. 1a). A single focal WT♀ or GA♀ and two males, one WT and one GA, were placed in a Petri dish (90 mm diameter, 15 mm height) with fresh distilled water in a 1.5 ml microcentrifuge tube and a pellet of rodent food (*n* = 25 WT♀ and 27 GA♀). To assay male choice, a single focal WT♂ or GA♂ was given a choice of two females, one WT♀ and one GA♀ (*n* = 27 WT♂ and 18 GA♂). Experiments were started using 0 day-old sexually unreceptive females and 10–12 days-old sexually mature males. Newly emerged (0 day-old) females were used to avoid the disruption of introducing a sexually mature female into the bioassay. *B. germanica* females become sexually receptive at 5–7 days of age, so the mating behavior of the focal insect was video-recorded for several days until they mated. Fertility of mated females was evaluated by the number of offspring produced. We assessed the gustatory phenotype of nymphs (either WT-type or GA-type) to determine which of the two adult cockroaches mated with the focal insect. Each gravid female was maintained individually in a container (95 × 95 × 80 mm) with food and water until the eggs hatched. Two day-old first instar nymphs were starved for one day without water and food, and then they were tested in Two-choice feeding assays using 1000 mmol l^−1^ glucose-containing agar with 0.5 mmol l^−1^ blue food dye vs. plain sugar-free agar with 1 mmol l^−1^ red food dye. If all the nymphs chose the glucose-containing agar, their parents were considered WT♂ and WT♀. When all the nymphs showed glucose-aversion, they were raised to the adult stage. Newly emerged adults were backcrossed with WT cockroaches, and their offspring were tested in the Two-choice assay. When the parents were both GA, 100% of the offspring exhibited glucose-aversion. When the parents were WT and GA, the offspring showed a 1:1 ratio of glucose-accepting and glucose-aversive behavior. Mate choice, mating success ratio and the number of offspring were analyzed statistically.

We conducted no-choice mating assay using the WT and GA strains (Fig. 1b, d). A female and a male were placed in a Petri dish with fresh water and a piece of rodent food and video-recorded for 24 h. The females were 5–7 days-old and males were 10–12 days-old. Four treatment pairs were tested: WT♂ × WT♀ (*n* = 20, 18 and 14 pairs for 5, 6 and 7 day-old females, respectively); GA♂ × GA♀ (*n* = 23, 22 and 35 pairs); GA♂ × WT♀ (*n* = 21, 14 and 17 pairs); and WT♂ × GA♀ (*n* = 33, 19 and 15 pairs).

To confirm that gustatory stimuli guide nuptial feeding, we artificially augmented the male nuptial secretion and assessed whether the duration of nuptial feeding and mating success of GA♀ were affected (Fig. 2c). Before starting the mating assay with 5 day-old GA♀, 10–12 days-old WT♂ were separated into three groups: A control group did not receive any augmentation; A water control group received distilled water with 1 mmol l^−1^ blue dye (+Blue); A fructose group received 3000 mmol l^−1^ fructose solution with blue dye (+Blue+Fru). Approximately 50 nl of the test solution was placed into the tergal gland reservoirs using a glass microcapillary. No-choice mating assays were carried out for 24 h. *n* = 20–25 pairs for each treatment.

We evaluated the association of short nuptial feeding (Fig. 1c) and the GA trait we conducted no-choice mating assays using females from the recombinant lines (Fig. 3c). Before starting each mating assay with 4 day-old females from the WT, GA and recombinant lines (WT_aa, GA_AA and GA_Aa), the EC_50_ for glucose was obtained by the instantaneous Acceptance-Rejection assay using 0, 10, 30, 100, 300, 1000 and 3000 mmol l^−1^ glucose (WT♀ and WT_aa♀, non-starved; GA♀, GA_AA♀ and GA_Aa♀, 1-day starved). After the Acceptance-Rejection assay, GA_Aa♀ were separated into two groups according to their sensitivity for rejecting glucose; the GA_Aa_high sensitivity group rejected glucose at 100 and 300 mmol l^−1^, whereas the GA_Aa_low sensitivity group rejected glucose at 1000 and 3000 mmol l^−1^. We paired these females with 10–12 days-old WT♂ (*n* = 15 WT_aa♀, *n* = 20 GA_AA♀, *n* = 20 GA_Aa_high♀ and *n* = 17 GA_Aa_low♀).

### Feeding bioassay

We conducted two feeding assays: Acceptance-Rejection assay and Consumption assay. The Acceptance-Rejection assay assessed the instantaneous initial responses (binary: yes-no) of cockroaches to tastants, as previously described^7,22,27^. Briefly, acceptance means that the cockroach started drinking. Rejection means that the cockroach never initiated drinking. The percentage of positive responders was defined as the Number of insects accepting tastants/Total number of insects tested. The effective concentration (EC_50_) for each tastant was obtained from dose-response curves using this assay. The Consumption assay was previously described^27^. Briefly, we quantified the amount of test solution females ingested after they started drinking. Females were observed until they stopped drinking, and we considered this a single feeding bout.

We used the Acceptance-Rejection assay and Consumption assay, respectively, to assess the sensitivity of 5 day-old WT♀ and GA♀ for accepting and consuming the WT♂ nuptial secretion (Fig. 2a, b). The secretion was diluted with HPLC-grade water to 0.001, 0.01, 0.03, 0.1, 0.3 and 1 male-equivalents/µl (*n* = 20 non-starved females each). The amount of nuptial secretion consumed was tested at 0.1 male-equivalents/µl in the Consumption assay (*n* = 10 each).

The Acceptance-Rejection assay was used to calculate the effective concentration (EC_50_) of glucose for females in the WT, GA and recombinant lines (Fig. 3a, b). A glucose concentration series of 0.1, 1, 10, 100 and 1000 mmol l^−1^ was tested with one-day starved 4-day old females (*n* = 65 GA_Aa♀, *n* = 50 GA_AA♀ and *n* = 50 GA♀) and non-starved females (*n* = 50 WT_aa♀ and *n* = 16 WT♀).

The effects of female saliva on feeding responses of 5 day-old WT♀ and GA♀ were tested using the Acceptance-Rejection assay (Fig. 4a). Freshly collected saliva of WT♀ and GA♀ was immediately used in experiments. Assays were prepared as follows: 3 µl of 200 mmol l^−1^ maltose or maltotriose were mixed with 3 µl of either HPLC-grade water or saliva of WT♀ or GA♀. The final concentration of each sugar was 100 mmol l^−1^ in a total volume of 6 µl. This concentration represented approximately the acceptance EC_70_ for WT♀ and GA♀^27^. Nuptial secretion (1 µl representing 10 male-equivalents) was mixed with 1 µl of either HPLC-grade water or saliva from WT♀ or GA♀, and 8 µl of HPLC-grade water was added to the mix. The final concentration of the nuptial secretion was 1 male-equivalent/µl in a total volume of 10 µl. This concentration also represented approximately the acceptance EC_70_ for WT♀ and GA♀ (Fig. 2a). The mix of saliva and either sugar or nuptial secretion was incubated for 300 s at 25 °C. Additionally, we tested the effect of only saliva in the Acceptance-Rejection assay. Either 1-day starved or non-starved females were tested with water only and then a 1:1 mixture of saliva and water. Saliva alone did not affect acceptance or rejection of stimuli. *n* = 20–33 females from each strain.

To evaluate whether salivary enzymes are involved in the hydrolysis of oligosaccharides, the contribution of salivary glucosidases was tested using the glucosidase inhibitor acarbose in the Acceptance-Rejection assay (Fig. 4b), as previously described^27^. We first confirmed that the range of 0–125 mmol l^−1^ acarbose in HPLC-grade water did not disrupt the acceptance and rejection of tastants. Test solutions were prepared as follows: 2 µl of either HPLC-grade water or saliva of GA♀ was mixed with 1 µl of either 250 µmol l^−1^ of acarbose or HPLC-grade water, then the mixture was added to 1 µl of 400 mmol l^−1^ of either maltose or maltotriose solution. The total volume was 4 µl, with the final concentration of sugar being 100 mmol l^−1^. For assays with nuptial secretion, 1 µl of either HPLC-grade water or saliva from 5 day-old GA♀ was mixed with 0.5 µl of either 250 µmol l^−1^ of acarbose or HPLC-grade water. This mixture was added to 0.5 µl of 10 male-equivalents of nuptial secretion (i.e., 20 male-equivalents/µl). HPLC-grade water was added for a total volume of 10 µl and a final concentration of 1 male-equivalent/µl. The mix of saliva and either sugars or nuptial secretion was incubated for 5 min at 25 °C. All test solutions contained blue food dye. Test subjects were 5 day-old GA♀ and 20–25 females were tested in each assay.

### Nuptial secretion and saliva collections

The nuptial secretion of WT♂ was collected by the following method: Five 10–12 days-old males were placed in a container (95 × 95 × 80 mm) with 5 day-old GA♀. After the males displayed wing-raising courtship behavior toward the females, individual males were immediately decapitated and the nuptial secretion in their tergal gland reservoirs was drawn into a calibrated borosilicate glass capillary (76 × 1.5 mm) under the microscope. The nuptial secretions from 30 males were pooled in a capillary and stored at −20 °C until use. Saliva from 5 day-old WT♀ and GA♀ was collected by the following method: individual females were briefly anesthetized with carbon dioxide under the microscope and the side of the thorax was gently squeezed. A droplet of saliva that accumulated on the mouthparts was then collected into a microcapillary (10 µl, Kimble Glass). Fresh saliva was immediately used in experiments.

### GC-MS procedures for analysis of sugars

Standards of D-( + )-glucose (Sigma-Aldrich), D-( + )-maltose (Fisher Scientific) and maltotriose (Sigma-Aldrich) were diluted in HPLC-grade water (Fisher Scientific) at 10, 50, 100, 500 and 1000 ng/µl to generate calibration curves. Samples were vortexed for 20 s and a 10 μl aliquot of each sample was transferred to a Pyrex reaction vial containing a 10 μl solution of 5 ng/μl sorbitol (≥98%) in HPLC-grade water as internal standard and dried under a gentle flow of N_2_ for 20 min.

Samples containing degradation products from nuptial secretions were prepared by adding 15 μl of HPLC-water to each sample in a 1.5 ml Eppendorf tube, vortexed for 30 s and centrifuged at 8000 rpm (5223 RCF) for 5 min to separate lipids from the water layer. The water phase was transferred to a reaction vial using a glass capillary. This procedure was repeated with the remaining lipid layer and the water layers were combined in the same reaction vial containing 10 μl of a solution of 5 ng/μl sorbitol and dried under N2 for 20 min.

For derivatization of sugars and samples, each reaction vial received 12 μl of anhydrous pyridine under a constant N_2_ flow, then vortexed and incubated at 90 °C for 5 min. Three μl of N-methyl-N-(trimethylsilyl)trifluoroacetamide (MSTFA; Sigma-Aldrich) was added to each reaction vial and centrifuged at 1000 rpm (118 RCF) for 2 min. Vials were incubated in a heat block at 90 °C for 1.5 hr and vortexed every 10 min for the first 30 min of incubation.

The total volume of sample was ~10 μl, and 1 μl was injected into the GC-MS (6890 GC coupled to a 5975 MS, Agilent Technologies, Palo Alto, CA). The inlet was operated in splitless mode (17.5 psi) at 290 °C. The GC was equipped with a DB-5 column (30 m, 0.25 mm, 0.25 μm, Agilent), and helium was used as the carrier gas at an average velocity of 50 cm/s. The oven temperature program started at 80 °C for 1 min, increased at 10 °C/min to 180 °C, then increased at 5 °C/min to 300 °C, and held for 10 min. The transfer line was set at 250 °C for 24 min, ramped at 5 °C/min to 300 °C and held until the end of program. The ion source operated at 70 eV and 230 °C, while the MS quadrupole was maintained at 200 °C. The MSD was operated in scan mode, starting after 9 min (solvent delay time) with a mass range of 33–650 AMU.

For GC-MS data analysis, the sorbitol peak area was obtained from the extracted ion chromatograms with m/z = 205, the sorbitol base peak. The area of peaks of glucose, maltose and maltotriose were obtained from the extracted ion chromatograms using m/z = 204, the base peak of the three sugars. The most abundant peaks of each sugar were selected for quantification^36^, and these peaks did not coelute with other peaks. Then, the peak areas of the three sugars were divided by the area of the respective sorbitol peak in each sample to normalize the data and to correct technical variability during sample processing. This procedure was performed to obtain the calibration curves and quantification of sugars in our experiments.

The results of sugar analysis using GC-MS are reported in Supplementary Figs. 1–4.

### Analysis of nuptial secretions

We focused the GC-MS analysis on glucose, maltose and maltotriose in WT♂ nuptial secretion (Fig. 4c). To quantify the time-course of saliva-catalyzed hydrolysis of WT♂ nuptial secretion to glucose, 1 µl of GA♀ saliva was mixed with 1 µl of 10 male-equivalents/µl. We incubated the mixtures for 0, 5, 10 and 300 s at 25 °C, and added 4 µl of methanol to stop the enzyme activity (*n* = 5 each treatment). Each sample contained the nuptial secretions of 5 males to obtain enough detectable amount of sugars. For the statistical analysis, the amounts of sugars were divided by 5 to obtain the amount of sugars in 1 male (1 male-equivalent). These amounts were also used for generating Fig. 4c and Supplementary Table 9. In calculations of the concentration of the three sugars (mmol l^−1^), the mass and volume of the nuptial secretion were measured using 70–130 male-equivalents of undiluted secretion of each strain (*n* = 3). The mass and volume of the nuptial secretion/male, including both lipid and aqueous layers, were approximately 30–50 µg and 40–50 nl. Because it was difficult to separate the lipid layer from the water layer at this small scale, we roughly estimated that the tergal reservoirs of the four cockroach lines had 30 nl of aqueous layer that contained sugars.

To quantify the time-course of saliva-catalyzed hydrolysis of maltose and maltotriose to glucose, 1 µl of GA♀ saliva was mixed with 1 µl of 200 mmol l^−1^ of either maltose or maltotriose (Fig. 4d, e). Incubation time points were 0, 5, 10 and 300 s at 25 °C and methanol was used to stop the enzyme activity. Controls without saliva were also prepared using HPLC-grade water instead of saliva and 300 s incubations. *n* = 5 for each treatment.

### Photomicroscopy

The photographs of the tergal glands and mouthparts (Fig. 5) were obtained using an Olympus Digital camera attached to an Olympus CX41 microscope (Olympus America, Center Valley, PA).

### Statistics and reproducibility

The sample size and number of replicates for each experiment are noted in the respective section describing the experimental details. In summary, the samples sizes were: Mating bioassays, *n* = 18–80; Feeding assays, *n* = 16–65; Sugar analysis, *n* = 5; Life history parameters, *n* > 14. All statistical analyses were conducted in R Statistical Software (v4.1.0; R Core Team 2021) and JMP Pro 15.2 software (SAS Institute Inc., Carey, NC). For bioassay data and sugar analysis data, we calculated the means and standard errors, and we used the Chi-square test with Holm’s method for post hoc comparisons, *t*-test, and ANOVA followed by Tukey’s HSD test (all α = 0.05), as noted in each section describing the experimental details, results, and in Supplementary Tables 1–11.

### Reporting summary

Further information on research design is available in the Nature Research Reporting Summary linked to this article.

## Supplementary information

Supplementary Information

Reporting Summary

## Data Availability

Data associated with this manuscript have been archived in Dryad Digital Repository (https://datadryad.org/stash/share/d-iwGk7WrCVFKAtShLn-yueu1ppeiwlOk7Uak5V1ZBo).
