# Supplementary material for: Rapid evolution of an adaptive taste polymorphism disrupts courtship behavior
Source: Commun Biol. 2022 May 12;5:450. doi: 10.1038/s42003-022-03415-8 (PMC9098494; doi:10.1038/s42003-022-03415-8)
Supplement: Supplementary file 1 — Supplementary Information [file 42003_2022_3415_MOESM1_ESM.pdf]

Supplementary Information for
Rapid evolution of an adaptive taste polymorphism disrupts courtship behavior

Ayako Wada-Katsumata*, Eduardo Hatano, Samantha McPherson, Jules Silverman and Coby Schal*

Department of Entomology and Plant Pathology and W.M. Keck Center for Behavioral Biology, North Carolina State University, Raleigh, North Carolina, 27695 United States of America

Corresponding Authors:
Email: akatsum@ncsu.edu
Email: coby@ncsu.edu

Supplementary Information contains:
Supplementary Figures 1-4
Supplementary Tables 1-11
Supplementary Reference

Supplementary Figures 1–4

Peaks of glucose, maltose and maltotriose did not coelute with peaks of other analytes in the chromatograms. For instance, a comparison of relative EIC and Total Ion Chromatogram (TIC) of a sample of five *B. germanica* tergal gland secretions treated with GA saliva for 300 sec showed no indication of multiple overlapping peaks over the peaks of glucose (Supplementary Fig. 1a), maltose (Supplementary Fig. 1b) and maltotriose (Supplementary Fig. 1c). We favored the quantification over EIC data to assure that the selected peaks derived only from the sugars in our protocol. Furthermore, mass spectra (MS) of library references and sugars in samples (Supplementary Fig. 2) matched, certifying the purity of peaks prior to calculation of calibration curves and quantification of sugars in samples.

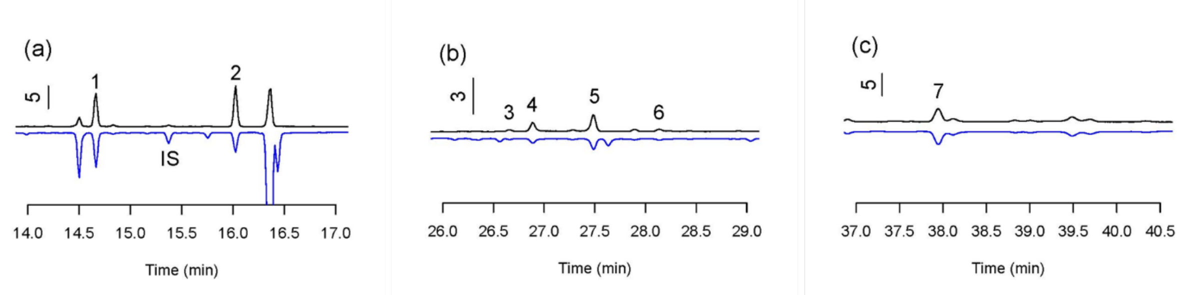

Supplementary Figure 1. Comparison of EIC (upper black traces; m/z = 204) and TIC (lower blue traces) of a sample of 5 gland contents treated with saliva for 300 sec. Chromatograms showing the peaks of (a) glucose (peaks 1 and 2), (b) maltose (peaks 3–6) and (c) maltotriose (peak 7). Relative chromatograms were calculated by dividing the EIC (m/z = 204) or TIC of each chromatogram by the sorbitol (IS) height (EIC by m/z = 205 or TIC height, respectively). Bars represent the relative intensity of chromatograms.

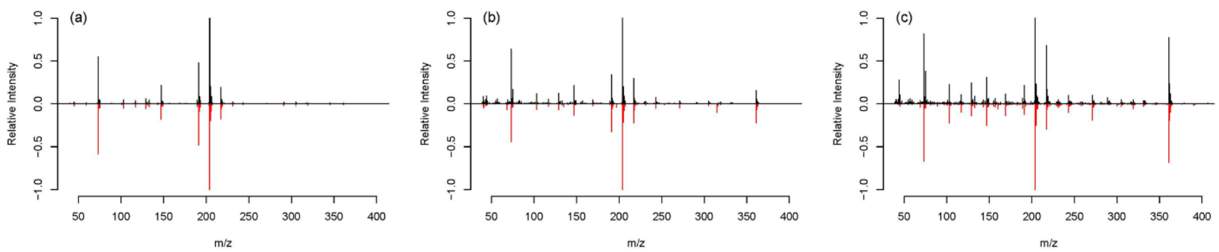

Supplementary Figure 2. Mass spectra (black traces) of (a) glucose, (b) maltose and (c) maltotriose in a sample of tergal secretion from 5 wild type glands treated with saliva for 300 sec mirrored against the respective reference mass spectra. Glucose and maltose MS references were obtained from the NIST library. The maltotriose reference was obtained from the GOLM Metabolome Database[1].

Each sample consisted of the tergal gland secretions of 5 males (5 male-equivalents/sample). Quantifications of glucose, maltose and maltotriose were performed by first normalizing the abundance of the peak of each sugar by the abundance of the internal standard in the sample, followed by fitting the relative abundances into the calibration curves (Supplementary Fig. 3) for final quantification. We calculated the amount of each sugar per male (1 male-equivalent) in each sample by dividing the GC-MS-derived amounts by 5. These data were used to conduct the statistical analysis and generate Figure 4c and Supplementary Table 9. All calibration curves had $R^2 < 0.97$. The tallest peak of each derivatized sugar was selected for quantification, following the protocol of Dhrami *et al.* (2011). Treatment with saliva over different periods of time did not affect the purity of peaks, which remained single peaks (Supplementary Fig. 4a-d). Glucose yielded two peaks (Supplementary Fig. 4b), maltose four peaks (Supplementary Fig. 4c) and maltotriose a single broad peak (Supplementary Fig. 4d). For maltotriose, a peak in the analyses of samples (Supplementary Fig. 4d; peak marked with an asterisk) matched the MS of derivatized maltotriose and used for quantification.

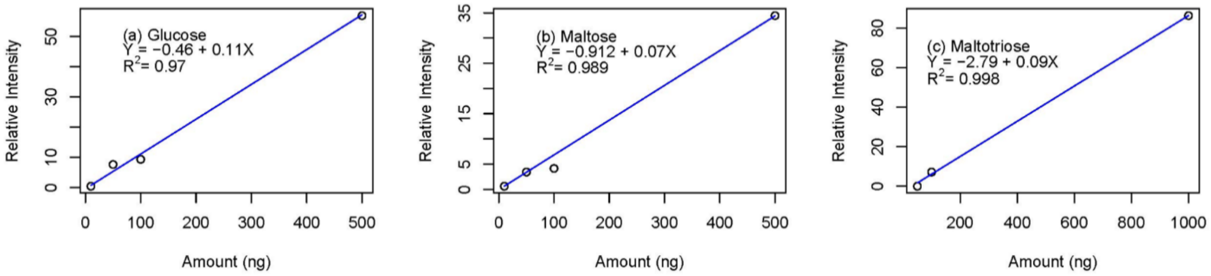

Supplementary Figure 3. Calibration curves of (a) glucose, (b) maltose and (c) maltotriose. Several concentrations of each carbohydrate (10, 50, 100 and 500 ng for glucose and maltose; 50, 100 and 1000 ng for maltotriose) were analyzed. Each dilution contained 50 ng sorbitol as internal standard (IS). Data points represent the relative peak intensity (respective peak area divided by the IS peak area). The regression line (blue) was fitted with the reverse order of carbohydrate amount as weights. Peak areas were estimated from extracted ion chromatograms (m/z = 205 for sorbitol; m/z = 204 for glucose, maltose and maltotriose). Linear equations and R^2 are displayed in each plot.

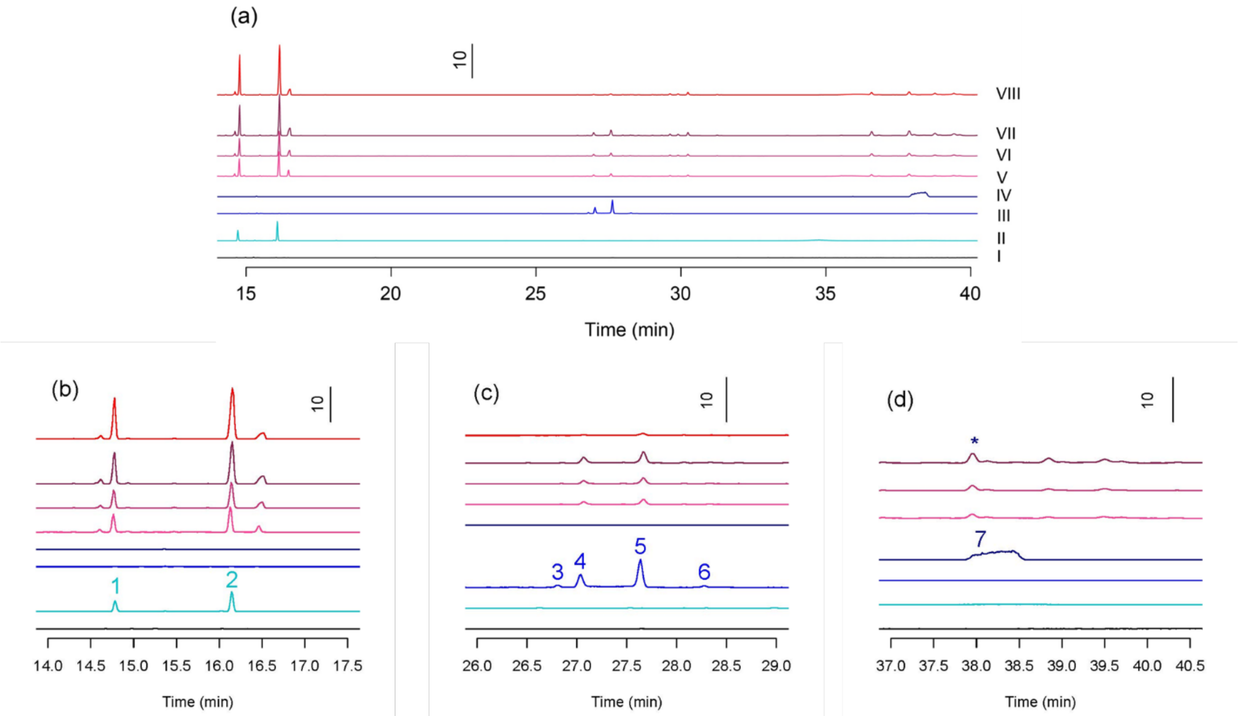

Supplementary Figure 4. Samples of the relative chromatograms of (I) sorbitol standard, (II) glucose standard, (III) maltose standard, (IV) maltotriose standard, (V) untreated tergal gland contents, (VI) gland contents treated with saliva for 5 sec, (VII) gland contents treated with saliva for 10 sec, and (VIII) gland contents treated with saliva for 300 sec. (a) Full chromatograms ranging from 14.7 to 40.5 min. (b–d) Sections of the chromatograms showing the peaks of (b) glucose (peaks 1 and 2), (c) maltose (peaks 3–6) and (d) maltotriose (peak 7). Peaks marked with numbers 2 and 5, and an asterisk were used for quantification of glucose, maltose and maltotriose, respectively. Relative chromatograms were calculated by dividing the EIC (m/z = 204) of each chromatogram by the sorbitol height (EIC by m/z = 205; IS). The vertical scale represents the relative intensity of chromatograms.

Supplementary Tables

Supplementary Table 1. Comparison of successful and failed courtship sequences in two-choice mating assays
Different letters indicate significant differences (α = 0.05)

% mating success						
Male	Female	Total (n)	Total mating success n (%)	Mating success with WT, n (%)	Mating success with GA, n (%)	Chi-square test, for mate choice
WT and GA	WT	25	25 (100.0) a	13 (52.0) a	12 (48.0) a	χ^2 (1) = 0.04, $p = 0.842$
WT and GA	GA	27	16 (59.3) b	4 (14.8) b	12 (44.4) a	χ^2 (1) = 4.00, $p =$ **0.046**
WT	WT and GA	31	31 (100.0) a	19 (61.3) a	12 (38.7) a	χ^2 (1) = 1.58, $p = 0.209$
GA	WT and GA	18	18 (100.0) a	12 (66.7) a	6 (33.3) a	χ^2 (1) = 2.00, $p = 0.157$
			Chi-square test, Holm χ^2 (3) = 33.83, $p <$ **0.001**	Letters compare treatments within rows		

Number of offspring							
Male	Female	Total (n)	Offspring with WT Mean ± SE % (n)		Offspring with GA Mean ± SE % (n)		t-test for mate choice
WT and GA	WT	25	40.5 ± 3.8 a	(13)	41.5 ± 4.2 a	(12)	$t = 0.91$, $p = 0.371$
WT and GA	GA	16	41.5 ± 1.3 a	(4)	40.7 ± 1.2 a	(12)	$t = -0.38$, $p = 0.712$
WT	WT and GA	31	39.2 ± 1.0 a	(15)	39.6 ± 0.7 a	(11)	$t = 0.66$, $p = 0.518$
GA	WT and GA	18	39.8 ± 0.9 a	(12)	40.7 ± 1.3 a	(6)	$t = 0.53$, $p = 0.607$
			Letters compare treatments within rows				

Supplementary Table 2. Comparison of successful and failed courtship sequences in no-choice mating assays

Different letters indicate significant differences (α = 0.05)

Number of pairs that mated or failed to mate							
	Total tested (*n*)	Not-mated group	Mated group				
Male × Female(Age)			In first encounter session	In last encounter session	Mating success (%)		Chi-square test, Holm
WT × GA(5-7)	67	33	3	31	34	50.7 b	χ^2 (3) = 16.70, *p* = **0.0008**
WT × WT(5-7)	52	9	5	38	43	82.7 a	
GA × WT(5-7)	52	12	0	40	40	76.9 a	
GA × GA(5-7)	80	30	0	50	50	62.5 ab	

% of pairs showing each behavior event in a single courtship sequence								
	Contact, %		Wing raising, %		Nuptial feeding, %		Copulation, %	
Male × Female(Age)	Failure	Success	Failure	Success	Failure	Success	Failure	Success
WT × GA(5-7)	100	100	100	100	100	97.4	0	100
WT × WT(5-7)	100	100	100	100	100	83.6	0	100
GA × WT(5-7)	100	100	100	100	100	98.0	0	100
GA × GA(5-7)	100	100	100	100	100	97.4	0	100

Frequency (# times) of each behavior in a single courtship sequence								
	Contact, frequency		Wing raising, frequency		Nuptial feeding, events		Copulation, frequency	
Male × Female(Age)	Failure	Success	Failure	Success	Failure	Success Mean ± SE	Failure	Success
WT × GA(5-7)	1.0	1.0	1.0	1.0	1.0	2.7 ± 0.5 a	0.0	1.0
WT × WT(5-7)	1.0	1.0	1.0	1.0	1.0	1.7 ± 0.2 b	0.0	1.0
GA × WT(5-7)	1.0	1.0	1.0	1.0	1.0	1.4 ± 0.1 b	0.0	1.0
GA × GA(5-7)	1.0	1.0	1.0	1.0	1.0	1.4 ± 0.1 b	0.0	1.0
ANOVA, Tukey's HSD test	-	-	-	-	-	*F* (3, 153) = 6.13, *p* = **0.0006**	-	-

Wing raising latency (sec), Mean ± SE		
Male × Female(Age)	Failure to mate sequence	Successful mating sequence
WT × GA(5-7)	6.6 ± 1.1	3.4 ± 0.4 ab
WT × WT(5-7)	5.6 ± 0.9	2.0 ± 0.2 b
GA × WT(5-7)	4.2 ± 0.4	3.1 ± 0.5 ab
GA × GA(5-7)	5.2 ± 0.6	3.7 ± 0.4 a
ANOVA, Tukey's HSD test	F (3, 239) = 1.607, $p = 0.19$	F (3, 163) = 4.007, $p =$ **0.009**

Nuptial feeding latency (sec), Mean ± SE		
Male × Female(Age)	Failure to mate sequence	Successful mating sequence
WT × GA(5-7)	773.7 ± 462.7	9.4 ± 1.7
WT × WT(5-7)	806.3 ± 646.9	6.6 ± 1.1
GA × WT(5-7)	52.1 ± 17.4	8.6 ± 1.7
GA × GA(5-7)	727.7 ± 328.3	7.4 ± 0.9
ANOVA	F (3, 236) = 0.68, $p = 0.57$	F (3, 153) = 0.78, $p = 0.50$

Nuptial feeding duration (sec), Mean ± SE				
Male × Female(Age)	Failure to mate sequence		Successful mating sequence	
	Nuptial duration	Total Nuptial duration	Nuptial duration	Total Nuptial duration
WT × GA(5-7)	2.8 ± 0.2 B	2.8 ± 0.2 B	3.8 ± 0.3 b	9.2 ± 1.4 ab
WT × WT(5-7)	4.6 ± 0.3 A	4.6 ± 0.3 A	6.9 ± 0.3 a	12.9 ± 1.6 a
GA × WT(5-7)	4.2 ± 0.2 A	4.2 ± 0.2 A	5.8 ± 0.3 a	7.6 ± 0.6 bc
GA × GA(5-7)	3.6 ± 0.2 B	3.6 ± 0.2 B	3.5 ± 0.2 b	4.8 ± 0.4 c
ANOVA, Tukey's HSD test	F (3, 236) = 12.53 $p <$ **0.0001**	F (3, 236) = 12.53 $p <$ **0.0001**	F (3, 153) = 31.66, $p <$ **0.0001**	F (3, 153) = 11.79, $p <$ **0.0001**

For Figure 1d

	Nuptial feeding duration (sec)	ANOVA, Tukey's HSD test
WT × WT(5-7) failed	4.6 ± 0.3 c	F (3, 167) = 18.97, $p <$ **0.0001**
GA × WT(5-7) failed	4.2 ± 0.2 c	
WT × WT(5-7) successful	6.9 ± 0.3 b	
GA × WT(5-7) successful	5.8 ± 0.3 a	

	Nuptial feeding duration (sec)	ANOVA, Tukey's HSD test
WT × GA(5-7) failed	2.8 ± 0.2 b	F (3, 222) = 3.69, $p =$ **0.01**
GA × GA(5-7) failed	3.6 ± 0.2 a	
WT × GA(5-7) successful	3.8 ± 0.3 a	
GA × GA(5-7) successful	3.5 ± 0.2 a	

Copulation latency (sec), Mean ± SE	
Male × Female(Age)	
WT × GA(5-7)	3.3 ± 0.5 ab
WT × WT(5-7)	3.6 ± 0.5 a
GA × WT(5-7)	3.3 ± 0.4 a
GA × GA(5-7)	2.2 ± 0.2 b
ANOVA, Tukey's HSD test	F (3, 153) = 3.10, p = **0.028**
**Copulation duration (sec), Mean ± SE**	
Male × Female(Age)	
WT × GA(5-7)	4,667 ± 88.5 b
WT × WT(5-7)	4,206 ± 108.0 b
GA × WT(5-7)	6,215 ± 140.1 a
GA × GA(5-7)	6,057 ± 145.3 a
ANOVA, Tukey's HSD test	F (3, 163) = 61.51, p < **0.0001**

Supplementary Table 3. Feeding responses (acceptance, rejection) of females to nuptial secretion

Concentration (male equivalents)	% feeding acceptance by WT♀ and GA♀of WT♂ nuptial secretion	
	WT♀ (n = 20)	GA♀ (n = 20)
0	0.0	0.0
0.01	30.0	30.0
0.03	55.0	55.0
0.1	75.0	70.0
0.3	85.0	85.0
1	100.0	100.0
EC_{50} (95% CI) (eq.)	0.021 (0.015, 0.027)	0.026 (0.018, 0.033)

Concentration (male equivalents)	Amount (µl) consumed by WT♀ and GA♀ of WT♂nuptial secretion		t-test
	WT♀ (n = 20)<br>Mean ± SE	GA♀ (n = 20)<br>Mean ± SE	
0.1	1.35 ± 0.21	0.33 ± 0.8	t = -4.56, p = **0.0002**

Supplementary Table 4. Effects of augmenting the nuptial secretion with fructose in no-choice mating assays

+B indicates the addition of a blue dye to the nuptial secretion. +B+Fru indicates the addition of 3000 mmol l^{-1} fructose with a blue dye to the nuptial secretion. Different letters indicate significant differences (α = 0.05)

Number of pairs that mated or failed to mate							
Male × Female (5 day-old) + treatment	Tested Total (*n*)	Not-mated	Mated				
			Success in first encounter	Success in other time point	Total (%)		Chi-square test, Holm
WT × GA	20	17	0	3	3	(15.0)	b
WT × GA +B	25	21	0	4	4	(16.0)	b χ^2 (2) = 13.23, p < **0.001**
WT × GA +B+Fru	20	8	0	12	12	(60.0)	a

Male × Female (5 day-old) + treatment	Mating failure and success sequences		
	Failure to mate sequence	Successful mating sequence	*t*-test
**Wing raising latency (sec), Mean ± SE**			
WT × GA	3.7 ± 0.6	2.3 ± 0.3	t = 0.82, p = 0.42
WT × GA +B	3.4 ± 0.3	2.3 ± 0.3	t = 1.64, p = 0.11
WT × GA +B+Fru	3.5 ± 0.4	2.6 ± 0.5	t = 1.56, p = 0.13
ANOVA	F (2, 60) = 0.17, p = 0.85	F (2, 16) = 0.106, p = 0.9	
**Nuptial feeding latency (sec), Mean ± SE**			
WT × GA	10.9 ± 1.8 b	5.7 ± 0.9	t = 1.20, p = 0.28
WT × GA +B	9.0 ± 1.8 b	5.0 ± 0.4	t = 0.85, p = 0.40
WT × GA +B+Fru	30.6 ± 10.5 a	11.6 ± 3.6	t = 1.43, p = 0.16
ANOVA, Tukey's HSD test	F (2, 60) = 4.52, p = **0.015**	F (2, 16) = 0.82, p = 0.46	
**Nuptial feeding duration (sec), Mean ± SE**			
WT × GA	2.3 ± 0.2 b	3.0 ± 0.0 b	t = -1.32, p = 0.20
WT × GA +B	2.7 ± 0.3 b	3.5 ± 0.3 b	t = -1.00, p = 0.33
WT × GA +B+Fru	4.3 ± 0.5 a	7.8 ± 0.5 a	t = -4.75, p < **0.0001**
ANOVA, Tukey's HSD test	F (2, 60) = 8.63, p = **0.0005**	F (2, 16) = 23.96, p < **0.0001**	
**Total duration of nuptial feeding events (sec), Mean ± SE**			
WT × GA	2.2 ± 0.2	7.7 ± 3.7	t = -4.00, p = **0.0006**
WT × GA +B	2.7 ± 0.4	7.3 ± 1.3	t = -4.49, p < **0.0001**
WT × GA +B+Fru	3.3 ± 1.0	8.8 ± 0.9	t = -4.67, p < **0.0001**
ANOVA, Tukey's HSD test	F (2, 41) = 1.24, p = 0.3	F (2, 16) = 0.32, p = 0.73	

Male × Female (5 day-old) + treatment	Mating failure and success sequences		
	Failure to mate sequence	Successful mating sequence	*t*-test
**Number of nuptial feeding events (times), Mean ± SE**			
WT × GA	1	2.7 ± 1.2	*t* = -4.19, *p* = **0.0004**
WT × GA +B	1	3.8 ± 1.0	*t* = -6.77, *p* < **0.0001**
WT × GA +B+Fru	1	1.2 ± 0.2	*t* = -1.24, *p* = 0.23
ANOVA	-	*F* (2, 16) = 6.97, *p* < **0.0001**	
**Copulation latency (sec), Mean ± SE**			
WT × GA	-	3.0 ± 1.2	-
WT × GA +B	-	2.8 ± 0.5	-
WT × GA +B+Fru	-	2.6 ± 0.7	-
ANOVA	-	*F* (2, 16) = 0.45, *p* = 0.96	-
**Copulation duration (sec), Mean ± SE**			
WT × GA	-	3786.0 ± 786.8	-
WT × GA +B	-	4177.5 ± 615.4	-
WT × GA +B+Fru	-	5323.0 ± 358.1	-
ANOVA	-	*F* (2, 16) = 2.51, *p* = 0.11	-

Supplementary Table 5. Effective concentration of glucose that elicits feeding acceptance in 50% of 4 day-old WT♀ and WT_aa♀ and feeding rejection in 50% of 4 day-old GA♀, GA_AA♀ and GA_Aa♀

Glucose acceptance and rejection by 4 day-old females				
Strains	Tested (*n*)	Response type	Pre-test condition	EC_{50} (95% CI) (mmol l^{-1})
WT	16	Glucose appetitive	Non-starved	100.0 (76.6, 123.4)
WT_aa	50	Glucose appetitive	Non-starved	89.9 (93.5, 106.4)
GA_Aa	65	Glucose aversive	1 day starvation with water	263.4 (212.7, 314.2)
GA_AA	50	Glucose aversive	1 day starvation with water	15.2 (11.6, 18.8)
GA	50	Glucose aversive	1 day starvation with water	2.2 (1.5, 2.9)

Supplementary Table 6. No-choice mating assays using 5 day-old females from the recombinant lines

Different letters indicate significant differences (α = 0.05)

No-choice bioassays							
Tested pairs of mated and not-mated groups							
Females paired with WT♂	Tested Total (*n*)	Not-mated	Mated				
			Success in first encounter	Success in other time point	Total (%)	Chi-square test, Holm	
WT_aa♀	15	0	0	15	15 (100)	a	
GA_Aa_low♀	17	5	0	12	12 (70.6)	b	χ^2 (3) = 8.45,
GA_Aa_high♀	20	8	0	12	12 (60.0)	b	*p* = **0.018**
GA_AA♀	20	8	0	12	12 (60.0)	b	

Nuptial feeding events of successful mating sequences		
Females paired with WT♂	Successful mating sequence	ANOVA, Tukey's HSD test
Nuptial feeding duration (sec), Mean ± SE		
WT_aa♀	6.3 ± 0.3 a	*F* (3, 47) = 10.42, *p* < **0.0001**
GA_Aa_low♀	5.8 ± 0.6 a	
GA_Aa_high♀	3.8 ± 0.3 b	
GA_AA♀	4.1 ± 0.3 b	
Total duration of nuptial feeding events (sec), Mean ± SE		
WT_aa♀	7.3 ± 0.4	*F* (3, 47) = 0.56, *p* = 0.645
GA_Aa_low♀	6.5 ± 0.8	
GA_Aa_high♀	6.3 ± 0.7	
GA_AA♀	6.6 ± 0.6	
Number of nuptial feeding events (times), Mean ± SE		
WT_aa♀	1.2 ± 0.1 b	*F* (3, 47) = 6.17, *p* = **0.001**
GA_Aa_low♀	1.2 ± 0.1 b	
GA_Aa_high♀	1.8 ± 0.2 ab	
GA_AA♀	2.0 ± 0.2 a	

Supplementary Table 7. Effect of saliva on feeding responses (acceptance, rejection) of WT♀ and GA♀

Different letters indicate significant differences (α = 0.05)

	% acceptance of sugars and nuptial secretion by WT♀ and GA♀ (*n*)				
Test solution	Starved WT♀		Starved GA♀		Chi-square test, Holm
Water	100.0	(20)	100.0	(20)	-
Water + GA Saliva	100.0	(20)	100.0	(20)	
Water + WT Saliva	100.0	(20)	100.0	(20)	
Test solution	Non-starved WT♀		Non-starved GA♀		Chi-square test, Holm
Water	0.0	(20)	0.0	(20)	-
Water + GA Saliva	0.0	(20)	0.0	(20)	
Water + WT Saliva	0.0	(20)	0.0	(20)	
Maltose + Water	95.5 a	(22)	90.9 a	(22)	χ^2 (5) = 64.22, *p* < **0.0001**
Maltose + GA Saliva	77.3 a	(22)	13.6 b	(22)	
Maltose + WT Saliva	72.7 a	(22)	13.6 b	(22)	
Maltotriose + Water	100.0 a	(20)	100.0 a	(22)	χ^2 (5) = 59.59, *p* < **0.0001**
Maltotriose + GA Saliva	95.0 a	(20)	36.4 b	(22)	
Maltotriose + WT Saliva	95.0 a	(20)	35.0 b	(20)	
WT_NupS + Water	100.0 a	(20)	93.9 a	(33)	χ^2 (5) = 68.94, *p* < **0.0001**
WT_NupS + GA Saliva	85.0 a	(20)	27.3 b	(33)	
WT_NupS + WT Saliva	85.0 a	(20)	30.0 b	(20)	

Supplementary Table 8. Involvement of salivary glucosidases in sugar degradation and sugar acceptance by GA♀

Different letters indicate significant differences (α = 0.05)

Test solution	% acceptance of sugars and nuptial secretion by GA♀ (*n*)		Chi-square test, Holm
Maltose	96.0 a	(25)	
Maltose + Acarbose	92.0 a	(25)	χ^2 (3) = 61.92,
Maltose + GA Saliva	8.0 b	(25)	$p <$ **0.0001**
Maltose + GA Saliva + Acarbose	84.0 a	(25)	
Maltotriose	100.0 a	(25)	
Maltotriose + Acarbose	100.0 a	(25)	χ^2 (3) = 52.94,
Maltotriose + GA Saliva	40.0 b	(25)	$p <$ **0.0001**
Maltotriose + GA Saliva + Acarbose	100.0 a	(25)	
WT_NupS	100.0 a	(20)	
WT_NupS + Acarbose	100.0 a	(20)	χ^2 (3) = 42.5,
WT_NupS + GA Saliva	30.0 b	(20)	$p <$ **0.0001**
WT_NupS + GA Saliva + Acarbose	90.0 a	(20)	

Supplementary Table 9. Time-course of saliva degradation of nuptial secretion
Different letters indicate significant differences (α = 0.05)

Glucose concentration (ng) after incubation of nuptial secretion with saliva of GA♀ (*n* = 5 for each treatment)		
Incubation time (sec)	WT♂ nuptial secretion<br>Mean ± SE	ANOVA,<br>Tukey's HSD test
0 (with saliva)	25.5 ± 0.7 c (4.7)	F (3, 16) = 64.2,<br>p < **0.0001**
5 (with saliva)	30.7 ± 2.0 c (5.7)	
10 (with saliva)	44.8 ± 2.5 b (8.3)	
300 (with saliva)	57.8 ± 1.5 a (10.7)	

Maltose concentration (ng) after incubation of nuptial secretion with saliva of GA♀ (*n* = 5 for each treatment)		
Incubation time (sec)	WT♂ nuptial secretion<br>Mean ± SE	ANOVA,<br>Tukey's HSD test
0 (with saliva)	10.1 ± 1.6 jk (1.0 mmol l^{-1})	F (3, 16) = 6.2,<br>p = **0.005**
5 (with saliva)	11.3 ± 1.0 jk (1.1 mmol l^{-1})	
10 (with saliva)	17.1 ± 3.8 j (1.7 mmol l^{-1})	
300 (with saliva)	4.1 ± 0.2 k (0.4 mmol l^{-1})	

Maltotriose concentration (ng) after incubation of nuptial secretion with saliva of GA♀ (*n* = 5 for each treatment)		
Incubation time (sec)	WT♂ nuptial secretion<br>Mean ± SE	ANOVA,<br>Tukey's HSD test
0 (with saliva)	17.2 ± 4.2 xy (1.1 mmol l^{-1})	F (3, 16) = 3.5,<br>p = **0.04**
5 (with saliva)	21.6 ± 2.4 xy (1.4 mmol l^{-1})	
10 (with saliva)	25.1 ± 1.9 x (1.7 mmol l^{-1})	
300 (with saliva)	13.4 ± 1.6 y (0.9 mmol l^{-1})	

Supplementary Table 10. Time-course of saliva degradation of maltose and maltotriose

Different letters indicate significant differences (α = 0.05)

	Amount of glucose released from incubation of maltose with 1 µl saliva from GA♀ (*n* = 5 for each treatment)	
Incubation time (sec)	Glucose (ng) Mean ± SE	ANOVA, Tukey's HSD test
0 (with saliva)	11.3 ± 1.5 b	F (4, 20) = 17.9, $p <$ **0.0001**
5 (with saliva)	23.4 ± 8.0 b	
10 (with saliva)	64.6 ± 19.7 b	
300 (with saliva)	226.6 ± 42.8 a	
300 (without saliva)	15.2 ± 4.7 b	

	Amounts of glucose and maltose released from incubation of maltotriose with 1 µl saliva from GA♀ (*n* = 5 for each treatment)			
Incubation time (sec)	Glucose (ng) Mean ± SE	ANOVA, Tukey's HSD test	Maltose (ng) Mean ± SE	ANOVA, Tukey's HSD test
0 (with saliva)	39.8 ± 2.2 b	F (4, 20) = 121.5, $p <$ **0.0001**	69.9 ±8.8 k	F (4, 20) = 51.1, $p <$ **0.0001**
5 (with saliva)	41.4 ± 2.1b		65.1 ±9.7 k	
10 (with saliva)	40.3 ±1.4 b		68.6 ±8.9 k	
300 (with saliva)	95.2 ±2.5 a		284.4 ±22.2 j	
300 (without saliva)	41.1 ±2.6 b		70.2 ±13.0 k	

Supplementary Table 11. Life history parameters of WT (Orlando Normal), GA (T-164), WT_aa and GA_aa cockroaches
Nine to 14 pairs were placed in individual cages to observe various parameters such as mating success, development of nymphs, and mortality. Different letters indicate significant differences between colonies (α = 0.05)

Event duration	Days				ANOVA, Tukey's HSD test or Chi-square test, Holm
			Recombinant lines		
	GA colony Mean ± SE (15/19 pairs)	WT colony Mean ± SE (14/14 pairs)	GA_AA Mean ± SE (8/10 pairs)	WT_aa Mean ± SE (9/9 pairs)	
Female emergence to Mating	5.3 ± 0.3	5.1 ± 0.3	4.8 ± 0.3	5.3 ± 0.2	F (3,41) = 0.99, p = 0.407
Mating to Egg hatch	22.9 ± 0.2 ab	22.3 ± 0.3 b	23.6 ± 0.2 a	23.7 ± 0.2 a	F (3,41) = 6.64, p = **0.002**
First to Second instar	5.3 ± 0.1 ab	4.9 ± 0.1 b	5.3 ± 0.2 ab	5.6 ± 0.2 a	F (3,41) = 4.00, p = **0.014**
Second to Third instar	5.5 ± 0.2	5.2 ± 0.2	5.4 ± 0.2	5.0 ± 0.0	F (3,41) = 1.18, p = 0.328
Third to Fourth instar	6.0 ± 0.3	5.4 ± 0.3	6.1 ± 0.2	5.9 ± 0.3	F (3,41) = 1.32, p = 0.283
Fourth to Fifth instar	6.7 ± 0.3	6.5 ± 0.2	6.6 ± 0.3	6.9 ± 0.2	F (3,41) = 0.47, p = 0.702
Fifth instar to Adult emergence	10.3 ± 0.5 a	9.1 ± 0.2 ab	8.8 ± 0.4 b	9.2 ± 0.3 ab	F (3,41) = 3.53, p = **0.023**
Other parameters					
Mating success (%)	78.9 b	100 a	80 b	100 a	χ^2 (3) = 17.50, p = **0.008**
Number of offspring	39. 6 ± 1.2	41.4 ± 1.4	43.3 ± 0.8	42.0 ± 0.7	F (3,41) = 2.49, p = 0.073
Number of adult females	17.7 ± 1.3 b	21.3 ± 1.3 a	21.9 ± 0.9 a	20.6 ± 0.9 a	F (3,41) = 4.01, p = **0.014**
Number of adult males	19.3 ± 1.2	18.4 ± 1.1	18.6 ± 0.9	19.9 ± 1.0	F (3,41) = 0.48, p = 0.697
Survivorship (%)	93.5 ± 1.8	95.9 ± 2.3	93.7 ± 1.6	96.3 ± 1.2	F (3,41) = 0.91, p = 0.446

Supplementary Reference

1. Schauer, N., Steinhauser, D., Strelkov, S., Schomburg, D., Allison, G., Moritz, T., Lundgren, K., Roessner-Tunali, U., Forbes, M. G., Willmitzer, L., Fernie, A. R. & Kopka, J. GC-MS libraries for the rapid identification of metabolites in complex biological samples. *FEBS Lett.* **579**, 1332-1337 (2005). doi: 10.1016/j.febslet.2005.01.029. (MS of derivatized maltotriose (11TMS) can be found at: gmd.mpimp-golm.mpg.de/Spectrums/1decff40-6d00-4102-9454-06d594d238d5.aspx)
