# Supplementary material for: Rapid evolution of an adaptive taste polymorphism disrupts courtship behavior
Source: Commun Biol. 2022 May 12;5:450. doi: 10.1038/s42003-022-03415-8 (PMC9098494; doi:10.1038/s42003-022-03415-8)
Supplement: Supplementary file 2 — Reporting Summary [file 42003_2022_3415_MOESM2_ESM.pdf]

nature portfolio

Corresponding author(s): Dr. Ayako Wada-Katsumata, Dr. Coby Schal

Last updated by author(s): Mar 25, 2022

Reporting Summary

Nature Portfolio wishes to improve the reproducibility of the work that we publish. This form provides structure for consistency and transparency in reporting. For further information on Nature Portfolio policies, see our Editorial Policies and the Editorial Policy Checklist.

Statistics

For all statistical analyses, confirm that the following items are present in the figure legend, table legend, main text, or Methods section.

n/a	Confirmed	
☐	☒	The exact sample size (n) for each experimental group/condition, given as a discrete number and unit of measurement
☐	☒	A statement on whether measurements were taken from distinct samples or whether the same sample was measured repeatedly
☐	☒	The statistical test(s) used AND whether they are one- or two-sided<br>*Only common tests should be described solely by name; describe more complex techniques in the Methods section.*
☒	☐	A description of all covariates tested
☐	☒	A description of any assumptions or corrections, such as tests of normality and adjustment for multiple comparisons
☐	☒	A full description of the statistical parameters including central tendency (e.g. means) or other basic estimates (e.g. regression coefficient) AND variation (e.g. standard deviation) or associated estimates of uncertainty (e.g. confidence intervals)
☐	☒	For null hypothesis testing, the test statistic (e.g. F, t, r) with confidence intervals, effect sizes, degrees of freedom and P value noted<br>*Give P values as exact values whenever suitable.*
☒	☐	For Bayesian analysis, information on the choice of priors and Markov chain Monte Carlo settings
☒	☐	For hierarchical and complex designs, identification of the appropriate level for tests and full reporting of outcomes
☒	☐	Estimates of effect sizes (e.g. Cohen's d, Pearson's r), indicating how they were calculated

Our web collection on statistics for biologists contains articles on many of the points above.

Software and code

Policy information about availability of computer code

Data collection	Video recording: The mating behavior of the focal insect was video-recorded for several days until they mated using an infra-red-sensitive camera (Polestar II EQ610, Everfocus Electronics) coupled to a data acquisition board and analyzed by searchable and frame-by-frame capable software (NV3000, AverMedia Information).<br>Sugar analysis: GC-MS (6890 GC coupled to a 5975 MS, Agilent Technologies, Palo Alto, CA)
Data analysis	Video analysis was done by software (NV3000, AverMedia Information)<br>Sugar analysis: Chemstation (Agilent)

For manuscripts utilizing custom algorithms or software that are central to the research but not yet described in published literature, software must be made available to editors and reviewers. We strongly encourage code deposition in a community repository (e.g. GitHub). See the Nature Portfolio guidelines for submitting code & software for further information.

Data

Policy information about availability of data

All manuscripts must include a data availability statement. This statement should provide the following information, where applicable:

- Accession codes, unique identifiers, or web links for publicly available datasets
- A description of any restrictions on data availability
- For clinical datasets or third party data, please ensure that the statement adheres to our policy

Published Dataset is available in https://doi.org/10.5061/dryad.t76hdr81k.
All raw and processed data will be made available upon request.

Field-specific reporting

Please select the one below that is the best fit for your research. If you are not sure, read the appropriate sections before making your selection.

☒ Life sciences ☐ Behavioural & social sciences ☐ Ecological, evolutionary & environmental sciences

For a reference copy of the document with all sections, see nature.com/documents/nr-reporting-summary-flat.pdf

Life sciences study design

All studies must disclose on these points even when the disclosure is negative.

Sample size	Mating bioassay: n = 18-80.<br>Individual feeding assay: n = 16-65.<br>Sugar analysis: n = 5.<br>Based on previous studies,we considered this sample size was sufficient for statistical analysis.
Data exclusions	Data were not excluded from analysis.
Replication	All replication attempts were successful and results were consistent.
Randomization	Insects used for bioassays were selected randomly under the same rearing conditions.<br>Nuptial secretions for sugar analysis were obtained from males randomly under the same rearing condition.
Blinding	Blinding was not possible as experimental conditions were evident from the data of bioassay and chemical analysis. Quantifications were performed using computational methods applied equally to all conditions and replicates.

Reporting for specific materials, systems and methods

We require information from authors about some types of materials, experimental systems and methods used in many studies. Here, indicate whether each material, system or method listed is relevant to your study. If you are not sure if a list item applies to your research, read the appropriate section before selecting a response.

Materials & experimental systems

n/a	Involved in the study	
☒	☐	Antibodies
☒	☐	Eukaryotic cell lines
☒	☐	Palaeontology and archaeology
☐	☒	Animals and other organisms
☒	☐	Human research participants
☒	☐	Clinical data
☒	☐	Dual use research of concern

Methods

n/a	Involved in the study	
☒	☐	ChIP-seq
☒	☐	Flow cytometry
☒	☐	MRI-based neuroimaging

Animals and other organisms

Policy information about studies involving animals; ARRIVE guidelines recommended for reporting animal research

Laboratory animals	The WT colony (Orlando Normal) was collected in Florida in 1947 and has served as a standard insecticide-susceptible strain. The GA colony (T-164) was also collected in Florida in 1989 and shown to be aversive to glucose; continued artificial selection with glucose-containing toxic bait fixed the homozygous GA trait in this population (approximately 150 generations as of 2020). To homogenize genetic backgrounds between the WT and GA strains, two recombinant colonies were initiated in 2013 by crossing 10 pairs of WT-males × GA-females and 10 pairs of GA-males × WT-females . At the F8 generation (free bulk mating without selection), 400 cockroaches were tested in Two-choice feeding assays that assessed their initial response to tastants, as described in previous studies. The cockroaches were separated into glucose-accepting and glucose-rejecting groups by the rapid acceptance-rejection assay (below). These colonies were bred for three more generations (F11 generation), and 200 cockroaches from each group were assayed and backcrossed to obtain homozygous glucose-accepting (aa) and glucose-averse (AA) lines. Similar results were obtained in both directions of the cross, confirming previous findings of no sex linkage of the GA trait. These two lines were defined as WT_aa (homozygotes, glucose-accepting) and GA_AA (homozygotes, glucose-averse). To obtain heterozygous GA cockroaches, GA_Aa, a single intercross group was generated from crosses of 10 pairs of WT_aa-males × GA_AA-females and 10 pairs of GA_AA-males × WT_aa-females.
Wild animals	No wild animals were used in this study.
Field-collected samples	No field-collected samples were used in this study.

Ethics oversight	The insects were reared and tested under appropriate conditions.

Note that full information on the approval of the study protocol must also be provided in the manuscript.
